# Supplementary material for: Epigen-mediated mechanisms to alleviate glucose homeostasis disruptions in diet-induced obese and STZ-induced diabetic mice
Source: Mol Ther. 2025 Jun 30;33(11):5775–90. doi: 10.1016/j.ymthe.2025.06.044 (PMC12628056; doi:10.1016/j.ymthe.2025.06.044)
Supplement: Document S1. Figures S1–S6 and Tables S1 and S2 [file mmc1.pdf]

YMTHE, Volume *33*

Supplemental Information

Epigen-mediated mechanisms to alleviate glucose homeostasis disruptions in diet-induced obese and STZ-induced diabetic mice

Ka-Ying Chan, Chu-Jun Deng, Dilun Chen, Tak-Ho Lo, Shiqi Jia, Pauline Po Yee Lui, and Chi-Ming Wong

Table S1 Baseline characteristics of study cohorts of normal or overweight/obese individuals. Data represent as mean ± SEM. Abbreviations: HbA1c, glycated haemoglobin, BMI, body mass index, SP, systolic pressures, DP, diastolic pressure, A/G, ALB/GLB ratio, ALB, albumin, GLB, globulin, ALP, alkaline phosphatase, ALT, alanine aminotransferase, AST, aspartate aminotransferase, GGT, Gamma-glutamyl transferase, DBIL, direct bilirubin, IBIL, indirect bilirubin, TBIL, total bilirubin.

	Normal	Overweight/Obese
Gender M/F	37/36	39/11
HbA1c (%)	5.8 ± 0.2	6.3 ± 0.2
epigen	274.3 ± 8.3	254.3 ± 10.3
Age (year)	48.7 ± 3.3	45.6 ± 2.4
**BMI (kg/m^2)**	22.5 ± 0.1	27 ± 0.3
SP (mmHg)	133.6 ± 2.1	134 ± 1.7
DP (mmHg)	77.9 ± 1.3	82.1 ± 1.5
Random glucose (mmol/L)	5.9 ± 0.3	7 ± 0.5
A/G	1.1 ± 0	1.2 ± 0
ALB (g/L)	52.1 ± 18.7	37.2 ± 0.9
GLB (g/L)	29.6 ± 1.4	30.7 ± 0.8
ALP (U/L)	97.6 ± 1.4	82.2 ± 5.5
ALT (U/L)	35.9 ± 5.4	41.1 ± 12.1
AST (U/L)	52.2 ± 11.1	46.5 ± 13.1
GGT (U/L)	85.8 ± 25.2	52.4 ± 9.4
DBIL (mmol/L)	20.9 ± 11.7	18.7 ± 14.2
IBIL (mmol/L)	14.5 ± 4.5	14.4 ± 4.9
TBIL (mmol/L)	33.6 ± 15	34 ± 20

Table S2 Baseline characteristics of study cohorts of normal or diabetic individuals. Data represent as mean ± SEM. Abbreviations: HbA1c, glycated haemoglobin, BMI, body mass index, SP, systolic pressures, DP, diastolic pressure, A/G, ALB/GLB ratio, ALB, albumin, GLB, globulin, ALP, alkaline phosphatase, ALT, alanine aminotransferase, AST, aspartate aminotransferase, GGT, Gamma-glutamyl transferase, DBIL, direct bilirubin, IBIL, indirect bilirubin, TBIL, total bilirubin.

	Normal	Diabetic
Gender M/F	44/24	12/8
**HbA1c (%)**	5.46 ± 0.045	8.3 ± 0.3
epigen	282.51 ± 8.59	240.1 ± 12.3
Age (year)	40.32 ± 2.29	63.1 ± 1.7
BMI (kg/m^2)	23.6 ± 0.41	25.9 ± 1
SP (mmHg)	132.31 ± 1.67	137.4 ± 3.5
DP (mmHg)	80.2 ± 1.21	81.5 ± 2
Random glucose (mmol/L)	5.46 ± 0.17	11.6 ± 1
A/G	1.04 ± 0.13	1.1 ± 0
ALB (g/L)	31.57 ± 1.74	45.3 ± 11.1
GLB (g/L)	33.4 ± 2.16	31.4 ± 1
ALP (U/L)	142.75 ± 33.47	107.9 ± 25.8
ALT (U/L)	111 ± 34.8	25.7 ± 2.6
AST (U/L)	150.58 ± 41.5	25.6 ± 3.3
GGT (U/L)	181.91 ± 54.01	99.8 ± 36.4
DBIL (mmol/L)	64.4 ± 29.04	2.8 ± 0.3
IBIL (mmol/L)	31.1 ± 9.99	7.5 ± 0.7
TBIL (mmol/L)	95.3 ± 39	10.3 ± 0.9

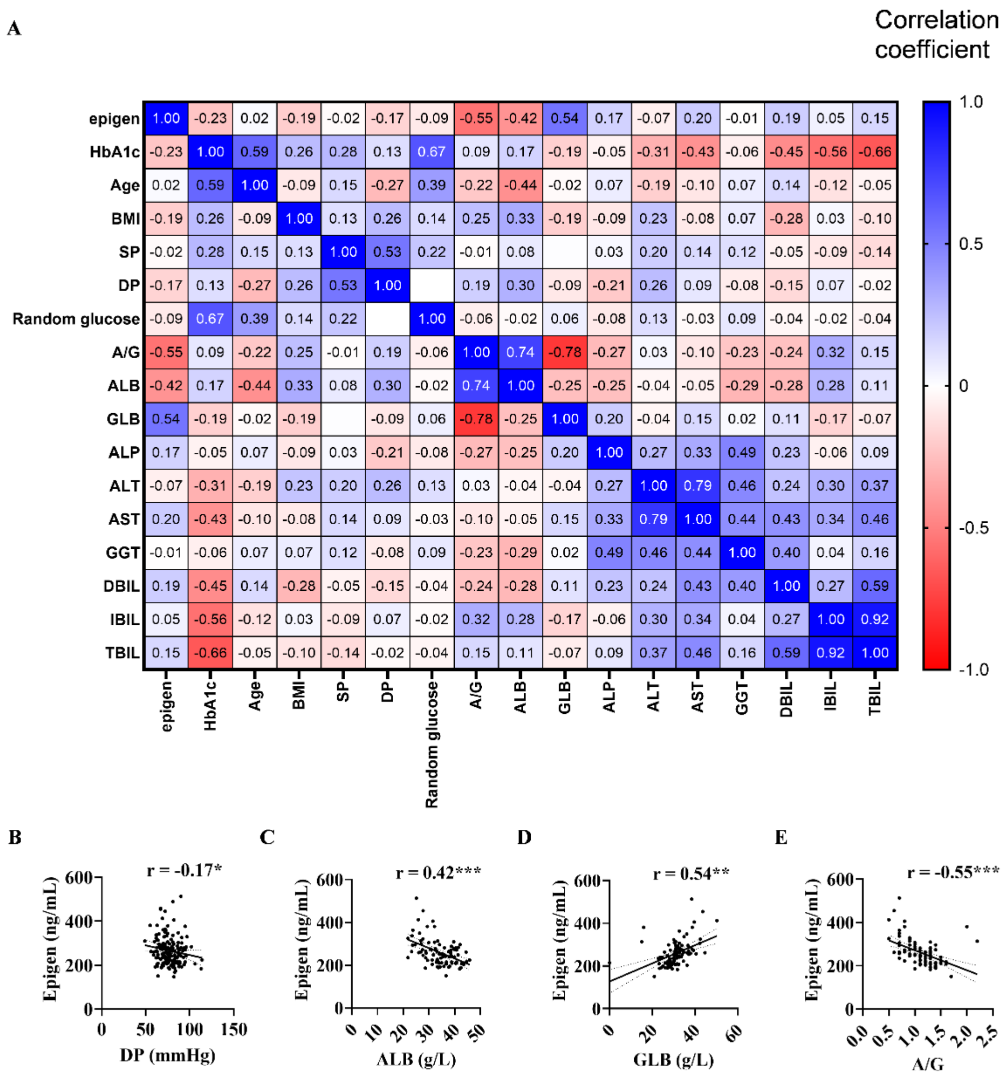

Figure S1 Correlations between circulating epigen levels and metabolic-related parameters in human serum samples. (A) Correlation matrix of epigen and metabolic-related parameters. Significant correlations between epigen and DP (B), ALB (C), GLB (D) and A/G (E). Statistical analysis was performed using Spearman's Rho Correlation. n = 159; ∗p < 0.05, ∗∗p < 0.01, ∗∗∗p < 0.001. Blank represent correlation coefficient < 0.01. Abbreviations: HbA1c, glycated haemoglobin, BMI, body mass index, SP, systolic pressures, DP, diastolic pressure, A/G, ALB/GLB ratio, ALB, albumin, GLB, globulin, ALP,

alkaline phosphatase, ALT, alanine aminotransferase, AST, aspartate aminotransferase, GGT, Gamma-glutamyl transferase, DBIL, direct bilirubin, IBIL, indirect bilirubin, TBIL, total bilirubin.

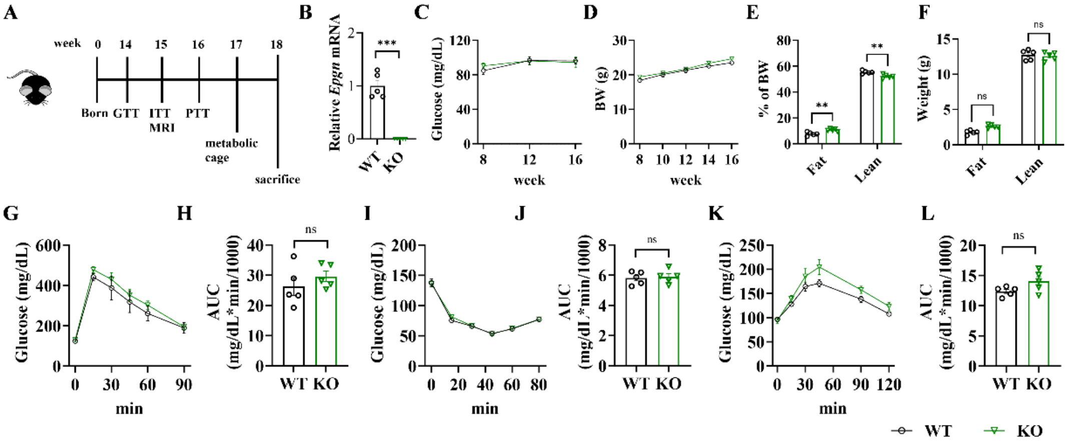

Figure S2 Glucose homeostasis in epigen knockout (KO) mice fed a standard chow diet. (A) Schematic diagram of experiment. (B) *Epgn* mRNA levels in the tongue. (C) Fasting glucose levels. Body weight measurements (D), fat and lean mass (E) and body composition (F) at 15 weeks. GTT results (G) and AUC (H) for the GTT at 14 weeks. ITT results (I) and AUC (J) for the ITT at 15 weeks. PTT results (K) and AUC (L) for the PTT at 16 weeks. n = 5 WT, 5 HFD. Data are the mean ± SEM. Statistical analysis was performed using unpaired two-tailed Student's t-test or Welch's t-test. ∗∗∗p < 0.001.

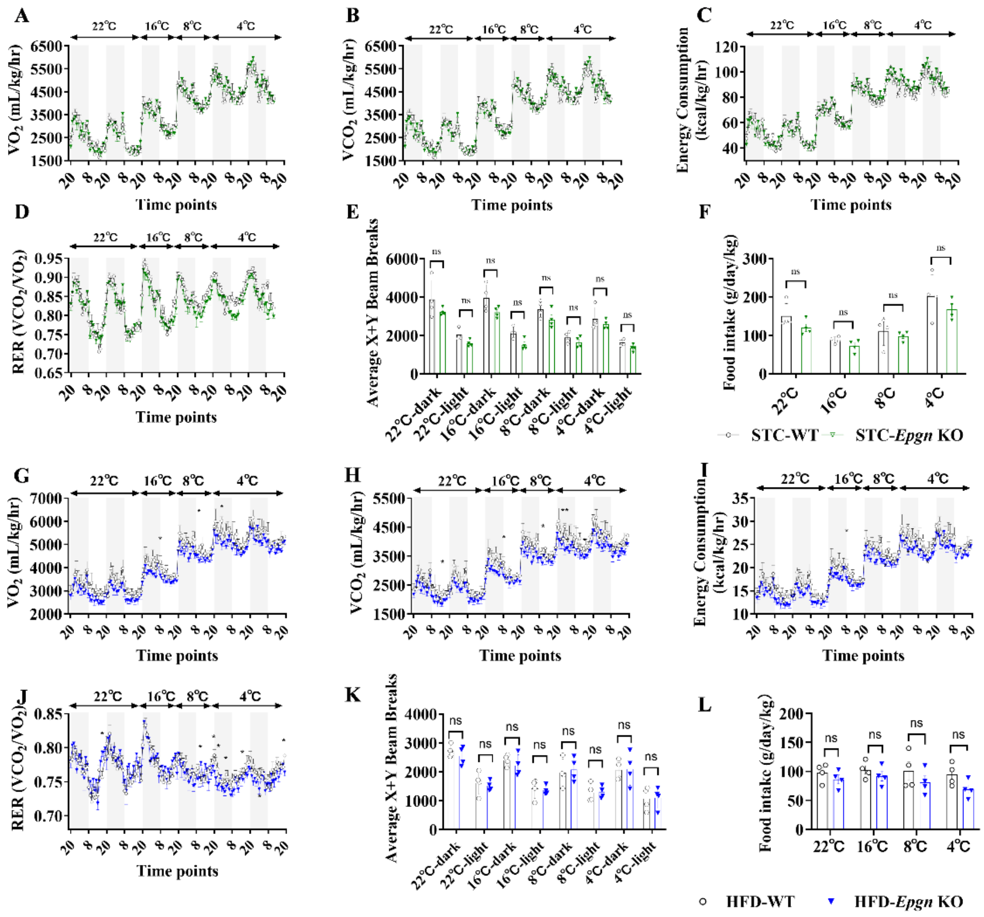

Figure S3 Energy expenditure in HFD-fed *Epgn* KO mice. Real-time monitoring curve of oxygen consumption (A), carbon dioxide release (B), energy consumption (C), respiratory exchange ratio (D) quantification of activity (E) and accumulated food intake (F) of STC-feeding 16 weeks old *Epgn* KO mice, comparing to their WT littermates. Real-time monitoring curve of oxygen consumption (G), carbon dioxide release (H), energy consumption (I), respiratory exchange ratio (J), quantification of activity (K) and accumulated food intake (L) of *Epgn* KO mice, comparing to their WT littermates after 12 weeks of HFD feeding. n = 4 WT, 4 KO. Data are the mean ± SEM. Statistical analysis was performed using unpaired two-tailed Student's t-test or Welch's t-test. $*p < 0.05$, $**p < 0.01$.

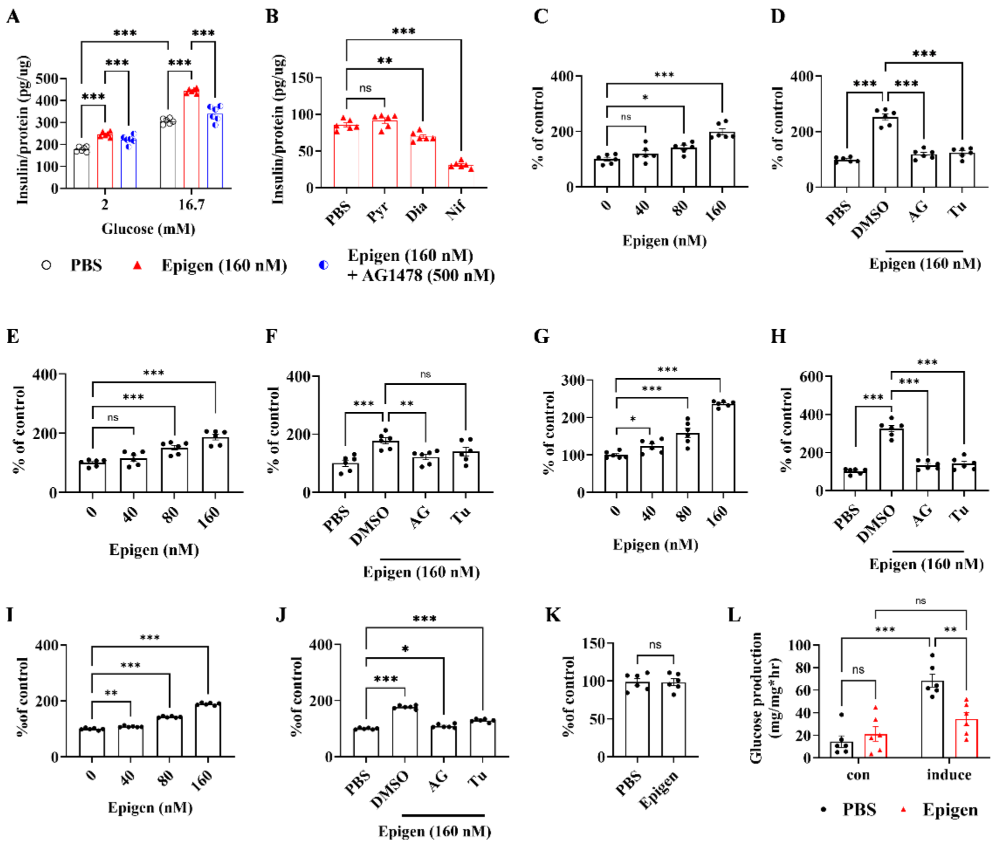

Figure S4 *In vitro* glucose uptake by cells and beta cell proliferation following acute recombinant epigen treatment. (A) Insulin secretion in SJ beta cells with epigen treatment under high glucose stimulation, with and without EGF receptor inhibitor AG1478. (B) Insulin secretion in SJ beta cells with epigen treatment, with and without calcium channel inhibitor Nifedipine (Nif). (C) Glucose uptake in 3T3-L1 differentiated adipocytes with epigen treatment in a dose-dependent manner. (D) Glucose uptake in 3T3-L1 differentiated adipocytes with and without EGF receptor inhibitors. (E) Glucose uptake in SW872 differentiated adipocytes with epigen treatment in a dose-dependent manner. (F) Glucose uptake in SW872 differentiated adipocytes with and without EGF receptor inhibitors. (G) Glucose uptake in L6 differentiated muscle cells with epigen treatment in a

dose-dependent manner. (H) Glucose uptake in L6 differentiated muscle cells with and without EGF receptor inhibitors. (I) Glucose uptake in A204 differentiated muscle cells with epigen treatment in a dose-dependent manner. (J) Glucose uptake in A204 differentiated muscle cells with and without EGFR inhibitors AG1478 and Tucatinib. (K) Glucose uptake in HepG2 cells with epigen treatment. (L) Glucose production in HepG2 cells under glucagon and pyruvate induction, with and without epigen protein treatment. Data are the mean ± SEM. Statistical analysis was performed using unpaired two-tailed Student's t-test or Welch's t-test. Group differences were determined via two-tailed analysis of variance (ANOVA) with Tukey's post hoc. $*p < 0.05$, $**p < 0.01$, $***p < 0.001$.

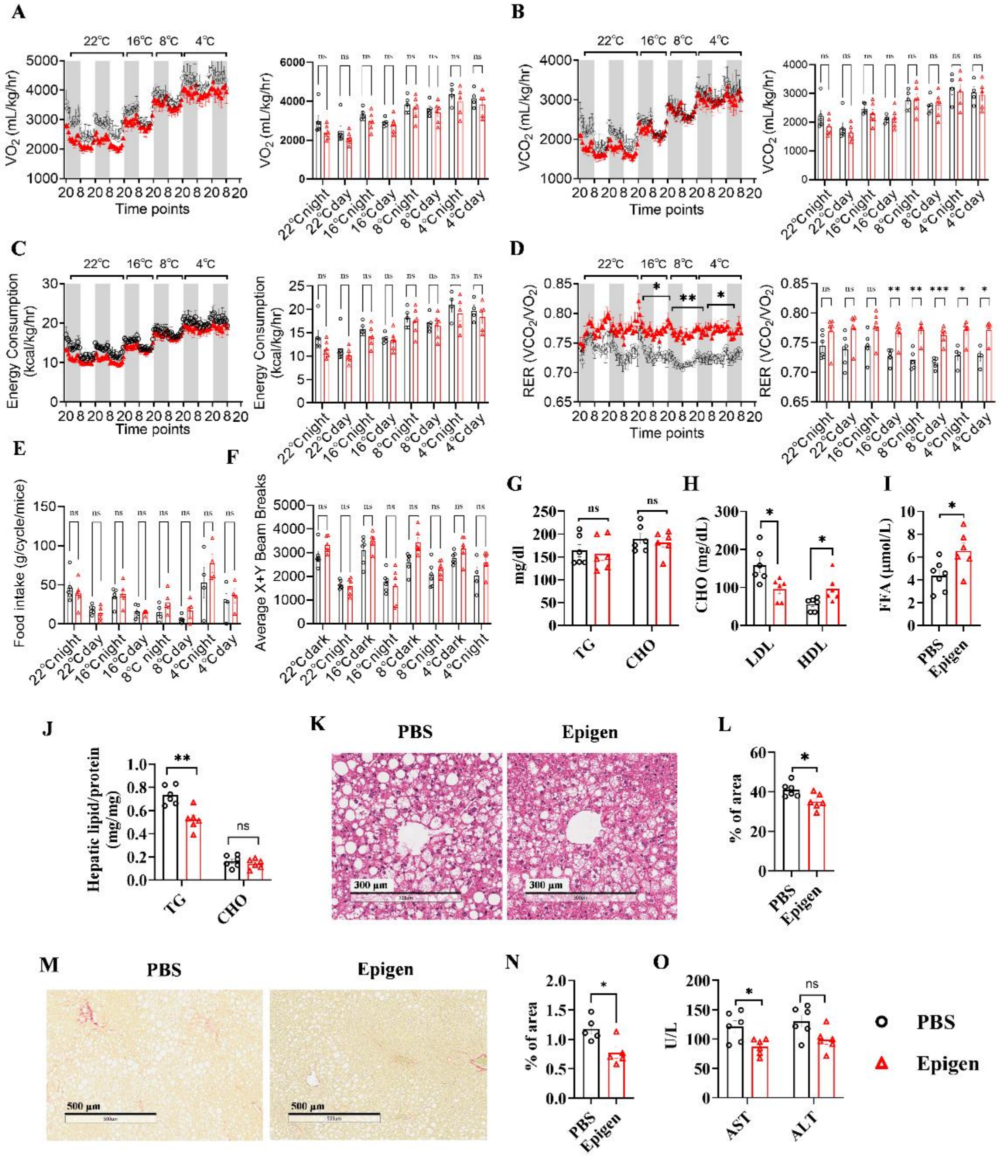

Figure S5 Energy expenditure and lipid profiles of mice after chronic epigen protein injection. (A) Real-time monitoring curve and average oxygen consumption (VO_2). (B) Real-time monitoring curve and averaged of carbon dioxide release (VCO_2). (C) Real-time monitoring curve and average energy consumption. (D) Real-time monitoring curve and average Respiratory exchange ratio (RER = VCO_2/VO_2). (E) Quantification of accumulated food intake. (F) Quantification of activity. (G) TG and CHO level. (H) LDL

and HDL level. (I) FFA level. (J) Hepatic lipid level. Representative images of liver H&E staining (K) and quantification of steatosis (L) Representative images of liver Sirius Red staining (M) and quantification of fibrosis (N) in liver. (O) AST and ALT levels at week 15. n = 6. Data are the mean ± SEM. Statistical analysis was performed using unpaired two-tailed Student’s t-test or Welch's t-test.

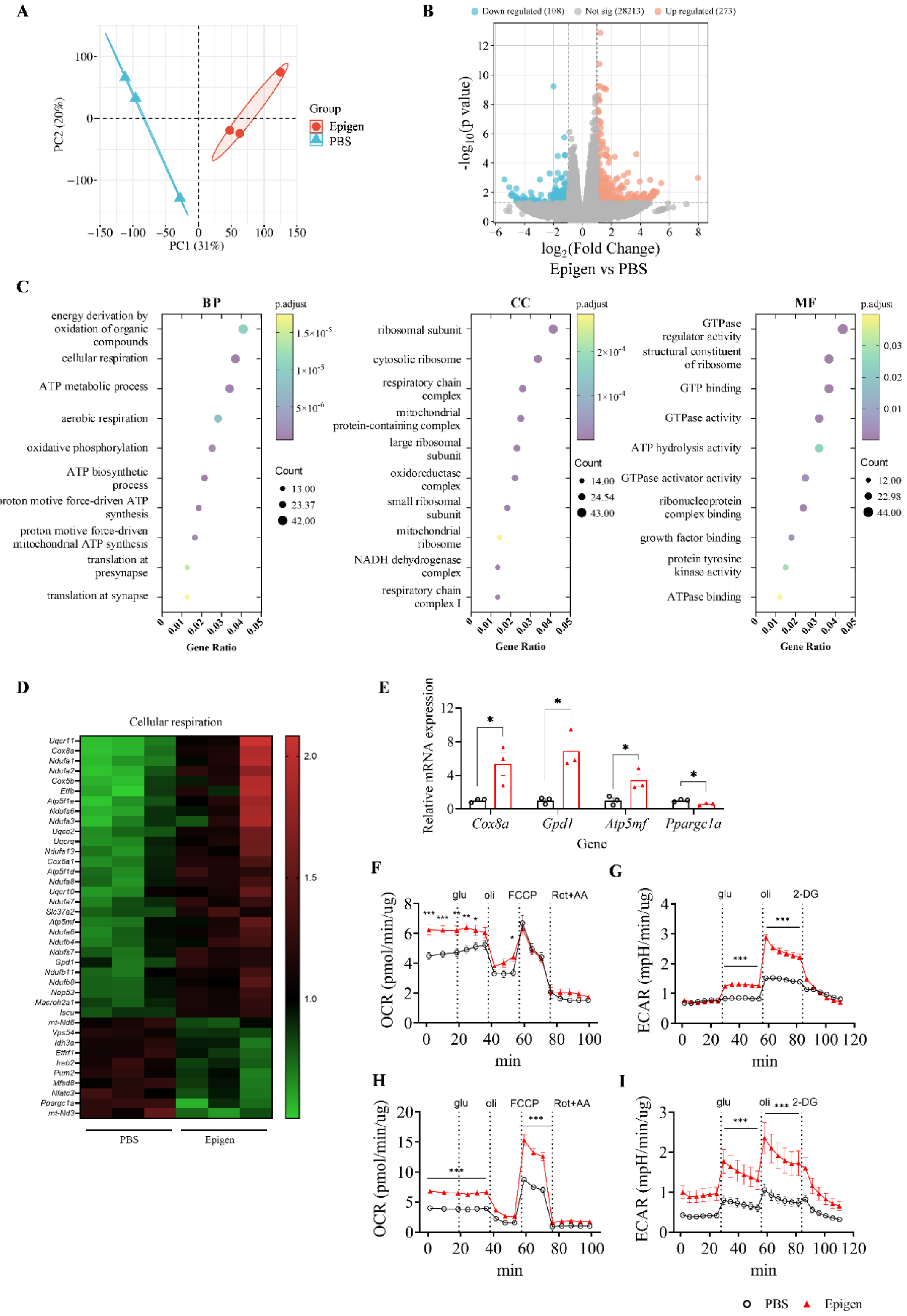
A
B
C
D
E
F
G
H
I
Group
Epigen
PBS
PC1 (31%)
PC2 (20%)
Down regulated (108)
Not sig (28213)
Up regulated (273)
Epigen vs PBS
BP
CC
MF
Gene Ratio
Cellular respiration
Relative mRNA expression
Gene
glu
oli
FCCP
Rot+AA
2-DG
OCR (pmol/min/ug)
ECAR (mpH/min/ug)
min
PBS
Epigen

Figure S6 RNA sequencing profile and functional enrichment analysis of differentially expressed genes (DEGs) in the sWAT of mice after 15 weeks of chronic epigen injection. (A) Principal component analysis (PCA) of samples. (B) Volcano plot representing sWAT DEGs. (C) Significantly enriched terms in Biological Processes (BP), Cellular Components (CC), and Molecular Functions (MF) from GO analysis. (D) Heatmap of gene expression related to the cellular respiration pathway. (E) RT-qPCR verification of the expression of select representative genes in sWAT. Measurements of oxygen consumption rate (OCR) of differentiated 3T3-L1 adipocytes (F) and differentiated SW872 adipocytes (H) with and without 160 nM epigen for 1 hour. Extracellular acidification rate (ECAR) of differentiated 3T3-L1 adipocytes (G) and differentiated SW872 adipocytes (I) with and without 160 nM epigen for 1 hour. A-E, n = 3; F, n = 8,9; G, n = 8,10; H, n = 10; I, n = 10,8. Data are the mean ± SEM. Statistical analysis was performed using unpaired two-tailed Student's t-test or Welch's t-test. $*p < 0.05$, $**p < 0.01$, $***p < 0.001$.
