# Supplementary material for: Epigen-mediated mechanisms to alleviate glucose homeostasis disruptions in diet-induced obese and STZ-induced diabetic mice
Source: Mol Ther. 2025 Jun 30;33(11):5775–90. doi: 10.1016/j.ymthe.2025.06.044 (PMC12628056; doi:10.1016/j.ymthe.2025.06.044)
Supplement: Document S2. Article plus supplemental information [file mmc2.pdf]

Molecular Therapy
Original Article

Epigen-mediated mechanisms to alleviate glucose homeostasis disruptions in diet-induced obese and STZ-induced diabetic mice

Ka-Ying Chan,[1] Chu-Jun Deng,[1] Dilun Chen,[2] Tak-Ho Lo,[1,3] Shiqi Jia,[4] Pauline Po Yee Lui,[5] and Chi-Ming Wong[1,6,7]

[1]Department of Health Informatics and Technology, The Hong Kong Polytechnic University, Hong Kong SAR, China; [2]Department of General Surgery Ward II, Foshan Sanshui District People's Hospital, Foshan, Guangdong Province, China; [3]School of Life Science, The Guangzhou University, Guangzhou, Guangdong Province, China; [4]Institutes of Biomedical Sciences, Inner Mongolia University, Hohhot, Inner Mongolia AR, China; [5]Department of Orthopaedics and Traumatology, Chinese University of Hong Kong, Hong Kong SAR, China; [6]State Key Laboratory of Pharmaceutical Biotechnology, University of Hong Kong, Hong Kong SAR, China; [7]Shenzhen Research Institute, The Hong Kong Polytechnic University, Shenzhen, China

Epidermal growth factor receptor (EGFR) plays a crucial role in cellular processes such as development and tissue repair, with dysregulation linked to various diseases, including those affecting energy metabolism. Previous studies have reported that EGFR ligands enhance glucose homeostasis through various mechanisms across different tissues. However, epigen, the latest EGFR ligand, has not been thoroughly investigated regarding its impact on energy metabolism. In this study, we employed both *in vivo* gain-of-function and loss-of-function approaches to investigate the role of epigen in metabolic regulation using diet-induced obesity (DIO) and streptozotocin (STZ)-induced diabetic mice. Our findings, which align with previous research on other EGFR ligands, provide the first evidence that epigen is crucial for glucose homeostasis. It promotes the release of endogenous insulin, enhances glucose uptake in adipose tissues and muscle, improves glycolysis and respiration utilization in adipose tissues of DIO mice, and increases pancreatic beta cell mass in STZ-induced diabetic mice. Overall, our findings suggest that epigen could be a potential therapeutic target for managing both type 2 and type 1 diabetes, warranting further exploration in clinical settings.

INTRODUCTION

The epidermal growth factor receptor (EGFR) is a member of the receptor tyrosine kinase (RTK) family of cell surface receptors. It is named after its interaction with the epidermal growth factor (EGF), which serves as its primary ligand. The binding of EGF to EGFR triggers a series of cellular signaling events that play a critical role in regulating cell growth, proliferation, and differentiation in various tissues and organs of the human body. Recent studies have indicated that EGFR activation also contributes to energy metabolism.[1–3] Currently, seven ligands bind to and activate the EGFR, namely EGF, transforming growth factor α (TGF-α), heparin-binding EGF-like growth factor (HB-EGF), betacellulin, amphiregulin, epiregulin, and epigen.[4] Among them, epigen is the last EGFR ligand most recently discovered in 2001 by high-throughput sequencing of a mouse keratinocyte complementary DNA library with homology to EGF family members.[5] Most EGFR ligands, except epigen, have been thoroughly investigated in terms of their impact on energy metabolism.[6–15]

Epithelial mitogen (epigen) is encoded by the *Epgn* gene located on Ensembl : chromosome 5: 91,175,323-91,183,074 in mice and EPGN on Ensembl : chromosome 4: 74,308,470-74,316,789 in humans. Both genes are next to the EGFR ligand amphiregulin locus.[16] Their transmembrane precursors share 79% identity and 85% similarity, and their mature active forms share 92% identity and 98% similarity. Two proteolytic cleavage events were required to release the segment harboring the EGF-like domain.[16] It was proposed that the membrane-flanking site of the epigen active form is cleaved by membrane-anchored metalloprotease ADM17[17] but the protease responses for cleavage at the N terminus remain to be identified.

Although epigen has been discovered for more than 20 years, its research and publication record remain very limited. Many of these publications merely mention epigen as one of the genes included in other omics studies. In fact, only a few of the publications have investigated the roles and functions of epigen.[5,17–26] In brief, unlike other EGFR ligands, epigen has low affinity and broad specificity[16] and it was proposed that epigen is mitogenic for fibroblasts and epithelial cells. Recombinant epigen could stimulate the proliferation of human epidermal keratinocyte HaCaT cells via c-erb-B1 receptors,[5] while knockdown epigen could reduce metastatic outgrowth in mouse and human breast cancer models.[18] However, the amount of epigen used to induce proliferation of HaCaT cells is 10- and

Received 24 January 2025; accepted 27 June 2025;
https://doi.org/10.1016/j.ymthe.2025.06.044.

Correspondence: Chi-Ming Wong, Department of Health Informatics and Technology, The Hong Kong Polytechnic University, Room Y945, 9/F, Lee Shau Kee Building, Hong Kong SAR, China.
E-mail: chi-ming.cm.wong@polyu.edu.hk

Published by Elsevier Inc. on behalf of The American Society of Gene and Cell Therapy.
This is an open access article under the CC BY license (http://creativecommons.org/licenses/by/4.0/).

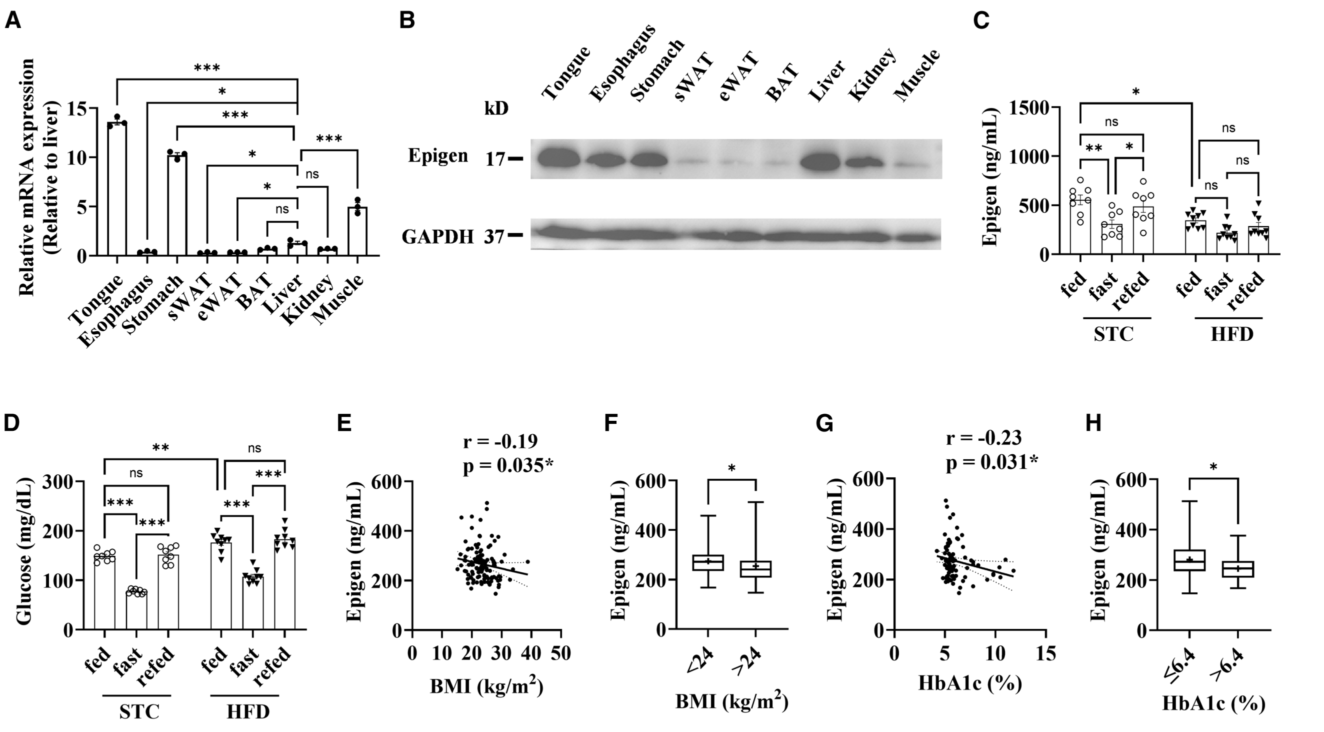

Figure 1. Circulating epigen levels in mice and humans

(A) *Epgn* mRNA levels (n = 3) and (B) epigen protein levels were measured in various mouse tissues. (C) Circulating epigen levels were assessed in mice during feeding, after a 16-h fast, and after 2 h of refeeding, with mice having been fed either STC or HFD for 6 weeks. (D) Corresponding glucose levels for (C) are also provided (n = 8 STC, 9 HFD). (E) A scatter correlation and (F) differential analysis box plot epigen levels and BMI in human serum samples (Table S1). (G) A scatter correlation and (H) differential analysis box plot for epigen levels and HbA1c levels in human serum samples (Table S2). Data are the mean ± SEM. Group differences were determined via two-tailed analysis of variance (ANOVA) with Tukey's post hoc test. Significance in correlations were determined with Spearman's rho. *$p < 0.05$, **$p < 0.01$, ***$p < 0.001$.

100-fold higher than the TGF-α and EGF, respectively.[23] However, more *in vivo* evidence is required to prove its pro-tumorigenic property and epithelial cell extrusion.

For *in vivo* studies, Dahlhoff et al. reported that transgenic mice overexpressing *Epgn* specifically in the skin had peripheral demyelination in 2013,[24] and sebaceous gland hyperplasia in 2014.[25] Interestingly, it was reported that recombinant epigen suppresses extrusion to provide protective effects against fungal invasion using zebrafish.[21] The phenotype of *Epgn* global knockout (KO) mice was first reported in 2013,[24] revealing that the mice are viable and do not exhibit any obvious phenotype. This means that the genetic deletion of *Epgn* does not affect mouse development, fertility, or organ physiology.[24] The authors proposed that it might be due to functional compensation by other EGFR ligands.[24] Despite these findings, the functions, and roles of *Epgn* remain largely unknown. Further research is needed to understand the specific mechanisms and effects of *Epgn* in various biological processes.

In this study, we comprehensively evaluated the role of epigen in energy metabolism, with a particular focus on glucose homeostasis, by both diet-induced obesity (DIO) and streptozotocin (STZ)-induced diabetic mouse models, along with *Epgn* KO mice and *Epgn* overexpressing mice using an adenovirus-mediated gene expression system. Furthermore, we also investigated the therapeutic potential of short- and long-term injections of epigen for treating diabetes, with findings that could inform the development of targeted therapies to treat diabetes.

RESULTS

Circulating epigen is tightly regulated by nutrient availability

First, we initially examined the expression of *Epgn* across various tissues. *Epgn* mRNA was mainly detected in the tongue, stomach, and muscle, with minimal expression in the other tissues examined (Cycle Threshold value > 35) via RT-qPCR (Figure 1A). Consistent with the mRNA expression pattern, epigen was also detected in these tissues, except muscle, where *Epgn* mRNA levels were high (Figure 1B). Notably, high levels of epigen were observed in the liver and kidney through western blotting (Figure 1B). As epigen functions as a hormone, it may be transported from the site of expression to other tissues via circulation. Then, we measured circulating epigen levels in mice at different feeding stages, comparing standard chow (STC) and high-fat diet (HFD) conditions (Figures 1C and 1D). Interestingly, under fed conditions, circulating epigen levels were higher in STC-fed mice compared with their HFD-fed littermates (Figure 1C). Specifically, during fasting, circulating epigen levels in STC-fed mice decreased

by 50%, a change not observed in HFD-fed mice (Figure 1C). In addition, circulating epigen levels in STC-fed mice, but not HFD-fed mice, varied in response to the fed-fast cycle (Figures 1C and 1D). Epigen was also detected in human circulation, with a mean level of 264.8 ng/mL and a standard error of the mean (SEM) of 5.1 ng/mL (Tables S1 and S2). Consistent with the mouse data described earlier, circulating epigen levels exhibited a significant negative correlation with BMI (Figures 1E and 1F) in the cohort of normal vs. overweight/obese subjects (Table S1), as well as with HbA1c levels (Figures 1G and 1H) in normal vs. diabetic subjects (Table S2). Interestingly, epigen levels also showed significant negative correlations with diastolic blood pressure (DBP) (Figure S1A) and albumin (ALB) (Figure S1B), along with positive correlations with globulin (GLB) (Figure S1C). Overall, the albumin-to-globulin ratio (A/G ratio) showed a significant negative correlation with epigen levels (Figure S1D).

Knockout of *Epgn* deteriorates metabolic dysregulation induced by HFD treatment

Given the change in the circulating levels of epigen by nutritional status (Figure 1) and its family members in regulation of energy metabolism,[6–15] we hypothesized that decrease in the circulating epigen levels may play a role in combating the pathogenesis of obesity. To assess the importance of epigen in metabolism, we conducted comprehensive metabolic phenotyping of *Epgn* KO mice (Figures 2A and S2). The validation of *Epgn* KO mice was confirmed through genotyping via PCR (Figures 2B and 2C), measurement of *Epgn* mRNA expression by RT-qPCR (Figure 2D), and assessment of circulating epigen levels by ELISA (Figure 2E).

Under STC conditions, *Epgn* KO mice did not exhibit any obvious metabolic phenotypes (Figure S2). Although, when subjected to HFD conditions (Figure 2), no statistically significant difference in body weight was observed between wild-type (WT) and *Epgn* KO mice (Figure 2G). Notably, *Epgn* KO mice displayed higher fasting glucose levels (Figure 2F) and increased fat mass (Figure 2H). The observed effects on net fat and lean mass resulted in a significant alteration in body composition, which was characterized by an increase in the percentage of fat mass and a decrease in the percentage of lean mass (Figure 2I). The increase in fat mass was primarily attributed to an increase in the mass (Figure 2J) and size of adipocytes in subcutaneous white adipose tissue (sWAT) and epididymal white adipose tissue (eWAT) (Figures 2K–2L). Increased immune cell infiltration in the WAT was also observed (Figure 2M).

Impaired glucose homeostasis in HFD-fed *Epgn* KO mice

HFD-fed *Epgn* KO mice exhibited impaired glucose tolerance, as shown by the glucose tolerance test (GTT; Figures 2N–2P), likely due to reduced glucose-stimulated insulin secretion (GSIS) (Figure 2Q). Insulin sensitivity, assessed by the insulin tolerance test (ITT), was comparable between *Epgn* KO and WT mice (Figures 2R–2T). *Epgn* KO significantly increased hepatic glucose production, as demonstrated by the pyruvate tolerance test (PTT) (Figures 2U–2W). The differences between GTT and PTT results were attributed to baseline glucose level variations, as normalizing the data to baseline eliminated these discrepancies (Figures 2P and 2W).

Impaired lipid homeostasis in HFD-fed *Epgn* KO mice

Then, we analyzed the lipid profiles of *Epgn* KO mice. Circulating triglyceride (TG) levels were unchanged (Figure 3A), but cholesterol (CHO) levels were elevated, primarily due to increased low-density lipoprotein (LDL) rather than high-density lipoprotein (HDL) (Figures 3B and 3C). Circulating free fatty acid (FFA) levels were lower in *Epgn* KO mice compared with WT controls (Figure 3D). Hepatic lipid analysis showed higher TG levels in *Epgn* KO mice, while CHO levels were similar to WT mice (Figures 3E and 3F). Liver lipid accumulation was greater in *Epgn* KO mice (Figures 3G and 3H). Despite increased hepatic lipid content, circulating liver damage markers aspartate aminotransferase (AST) and alanine aminotransferase (ALT) remained comparable between *Epgn* KO and WT mice (Figure 3I).

We also used metabolic cages to assess energy expenditure and food intake at various environmental temperatures. Although, under STC conditions, *Epgn* KO mice did not exhibit any obvious metabolic phenotypes as compared with their WT littermates (Figures S3A–S3F), there was a trend indicating that *Epgn* KO mice had lower VO_2 consumption (Figure S3G), VCO_2 production (Figure S3H), energy consumption (Figure S3I), and respiratory exchange ratio (RER; Figure S3J) for several data points under HFD conditions. Taken together, these findings suggest that the observed differences in *Epgn* KO mice may be due to intrinsic changes in lipid metabolism, potentially linked to lower energy expenditure that was not attributed to locomotor activity (Figure S3K) or the amount of food consumed (Figure S3L).

Overexpression of EPGN alleviates metabolic dysregulation induced by HFD treatment

As *Epgn* knockout deteriorates dysregulation of glucose homeostasis (Figure 2), we further evaluated the impact of epigen on metabolism by overexpressing *Epgn* in DIO mice using an adenovirus-mediated *Epgn* expression system (Figure 4A). Based on our experience, the liver serves as an effective metabolic organ for increasing hormone levels while minimizing obvious side effects.[27] The overexpression was validated by measuring *Epgn* mRNA levels in the liver via RT-qPCR (Figure 4B), hepatic epigen levels through western blotting (Figure 4C), and circulating epigen levels by ELISA (Figure 4D). Overexpression of *Epgn* did not affect body weight (Figure 4E) or fasting glucose levels (Figure 4F). However, improvements in glucose and pyruvate tolerance were observed, as indicated by the GTT (Figures 4G and 4H) and PTT (Figures 4J and 4K). There was no change in insulin sensitivity and secretion, as demonstrated by the ITT (Figures 4L and 4M) and GSIS (Figure 4I).

Acute injection of high-dosage epigen improves glucose homeostasis

To evaluate whether epigen injection mimics the effects of *Epgn* overexpression, we administered 10 mg/kg epigen intraperitoneally

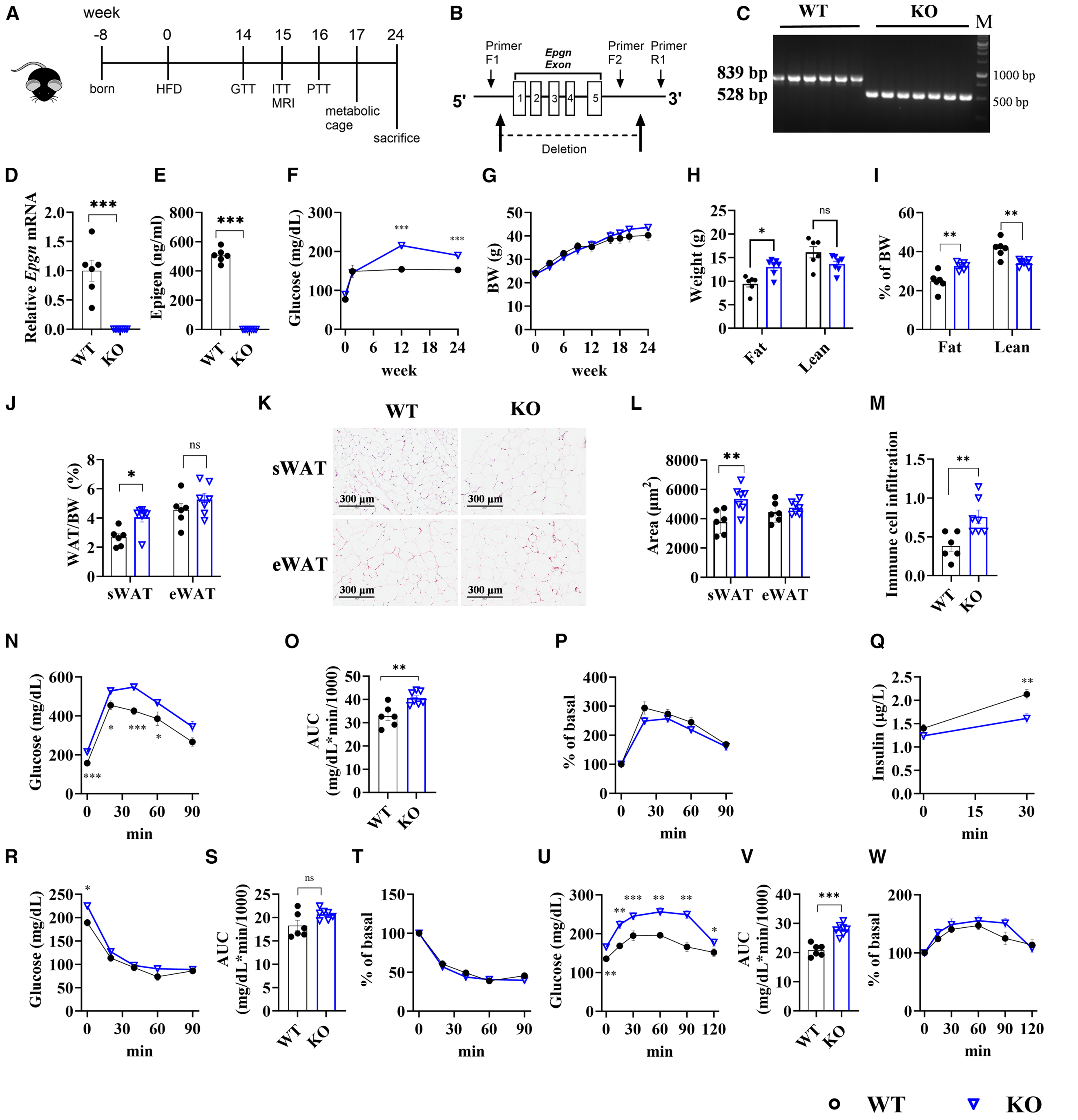

Figure 2. Glucose homeostasis in *Epgn* KO mice on an HFD

(A) Schematic diagram of the experiment. (B) Schematic diagram of the *Epgn* knockout and genotyping strategy. (C) Genotyping results using PCR with genomic DNA and the relative positions for the primers used specified in (B). (D) *Epgn* mRNA levels in the tongue. (E) Circulating levels of epigen. (F) Fasting glucose levels. (G) Body weight measurements. (H) Fat and lean mass measurements. (I) Percentage of fat and lean mass relative to body weight. (J) Percentage of sWAT and eWAT mass relative to body weight. (K) H&E staining of sWAT and eWAT. (L) Quantification of adipocyte size in sWAT and eWAT. (M) Quantification of immune cell infiltration in sWAT. (N) GTT results after 14 weeks of HFD feeding. (O) AUC, (P) percentage of basal glucose levels, and (Q) GSIS from the GTT in (M). (R) ITT results after 15 weeks of HFD feeding. (S) AUC and (T) percentage of basal glucose levels from the ITT in panel Q. (U) PTT results after 16 weeks of HFD feeding. (V) AUC and (W) percentage of basal glucose levels from the PTT in (T). n = 6 WT, 7 KO. Data are the mean ± SEM. Statistical analysis was performed using unpaired two-tailed Student's t test or Welch's t test. $^{*}p < 0.05$, $^{**}p < 0.01$, $^{***}p < 0.001$.

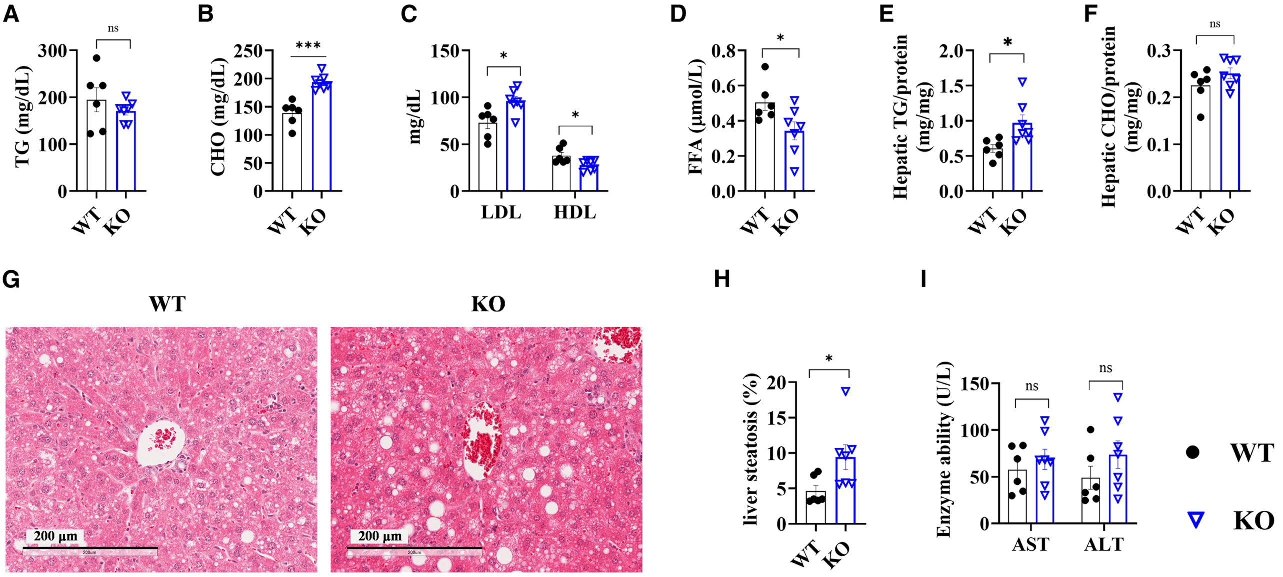

Figure 3. Lipid metabolism in global *Epgn* knockout (KO) mice

(A) Circulating TG level, CHO level (B), LDL and HDL levels (C), and FFA levels (D) after 6 months of HFD feeding. Hepatic TG (E) and CHO (F) levels in liver after 6 months of HFD feeding. Representative H&E staining images (G) and quantification of liver changes (H) after 6 months of HFD feeding. (I) Serum levels of AST and ALT after 6 months of HFD feeding. $n = 6$ WT, 7 KO. Data are the mean ± SEM. Statistical analysis was performed using unpaired two-tailed Student's t test or Welch's t test. *$p < 0.05$, **$p < 0.01$, ***$p < 0.001$.

to HFD-fed mice. Epigen improved glucose tolerance, as shown by GTT, compared with PBS-treated controls (Figures 5A and 5B). This effect was accompanied by increased circulating insulin levels (Figure 5C), suggesting an insulin-dependent glucose-lowering mechanism. Interestingly, epigen also enhanced glucose tolerance in STZ-treated mice (Figures 5D and 5E) without affecting circulating insulin levels (Figure 5F), indicating an additional insulin-independent glucose-lowering mechanism.

Epigen induces insulin secretion from mouse pancreatic beta cells

Given that acute epigen injection increased circulating insulin levels in HFD-fed mice (Figures 5A–5C), we explored whether epigen could induce insulin secretion from pancreatic beta cells by *ex vivo* and *in vitro* experiments. Epigen stimulated insulin secretion from mouse islets and SJ beta cells in a dose-dependent manner, with a stronger effect at high glucose levels (16.7 mM) compared with low glucose levels (2 mM; Figures 5G and S4A). This effect was blocked by the EGFR inhibitor AG1478 (Figures 5G and S4A) and the calcium channel inhibitor Nifedipine (Nif; Figures 5H and S4B). This is consistent with previous findings showing that activation of EGFR can increase calcium influx through voltage-gated calcium channels, a process critical for the exocytosis of insulin granules in pancreatic beta cells.[12,28]

Epigen promotes glucose uptake by adipocytes and muscle cells and represses glucose production by hepatocytes

As acute injection of epigen lowered glucose levels in STZ-treated mice an in insulin-independent manner (Figures 5D–5F), we explored whether epigen can directly induce glucose uptake by metabolic tissues. To determine the target organs through which epigen regulates glucose homeostasis, we utilized fluorescent labeling of epigen along with a fluorescent glucose tracer, 2-N-7-nitrobenz-2-oxa-1,3-diazol-4-ylamino-2-deoxyglucose (2-NBDG), followed by imaging with an *in vivo* fluorescence imaging (IVIS) Spectrum system. The labeled epigen accumulated in all examined organs (Figure 5I). Intriguingly, treatment with exogenous recombinant epigen promoted the uptake of 2-NBDG by eWAT (Figure 5J).

To evaluate whether epigen directly enhances glucose uptake in adipose tissues, we performed *in vitro* glucose uptake assays using adipocytes differentiated from mouse stromal vascular fraction (SVF), mouse 3T3-L1 cells, and human SW872 cells. Recombinant epigen significantly increased glucose uptake in a dose-dependent manner (Figures 5K, S4C, and S4E), and this effect was abolished by EGFR inhibitors, AG1478 and tucatinib (Figures 5L, S4D, and S4F). Since muscle tissue plays a critical role in glucose uptake during GTT, we also assessed the effect of epigen on muscle cells. Epigen enhanced glucose uptake in a dose-dependent manner in myoblast-differentiated mouse primary muscle cells (Figure 5M), rat L6-differentiated muscle cells (Figure S4G), and human A204-differentiated muscle cells (Figure S4I), with these effects similarly blocked by EGFR inhibitors (Figures 5N, S4H, and S4J). Consistent with IVIS imaging showing no significant 2-NBDG accumulation in the liver (Figure 5J), epigen treatment did not enhance glucose uptake in mouse primary hepatocytes (Figure 5O) or the human liver cell line HepG2 (Figure S4K).

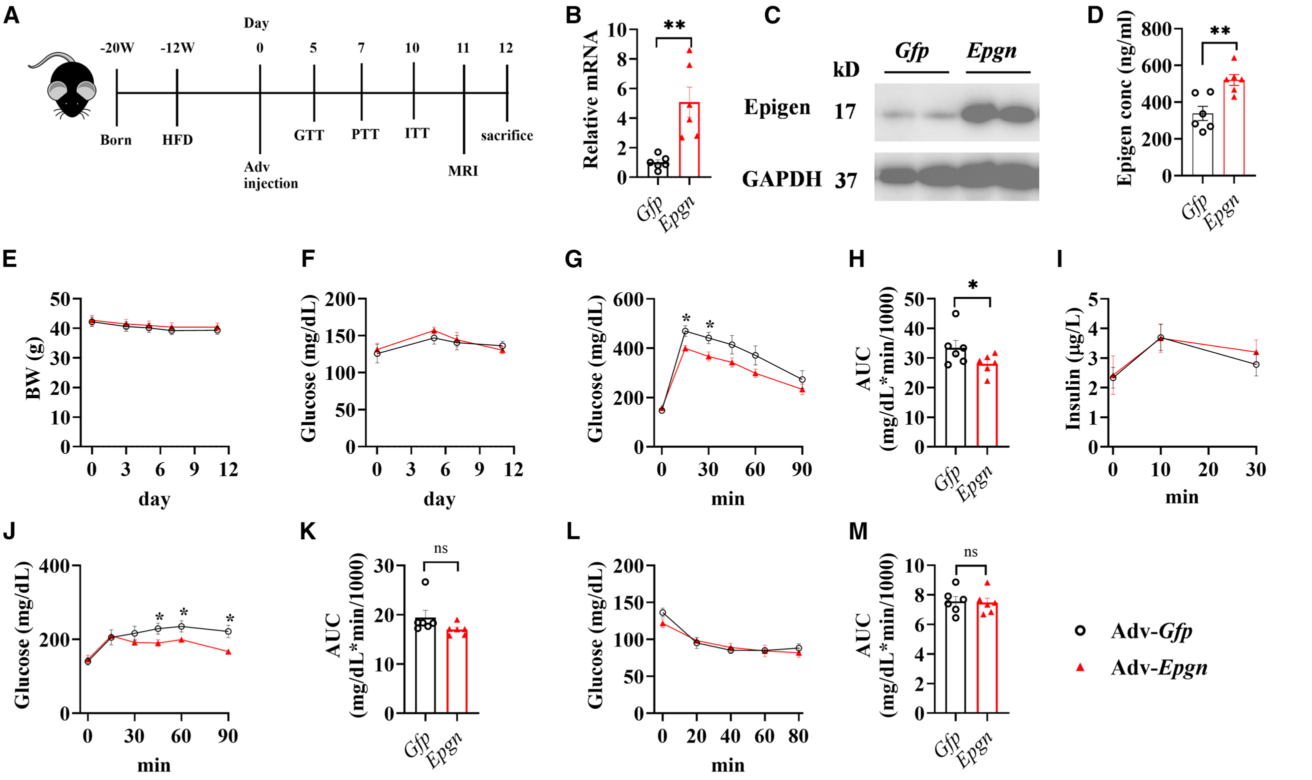

Figure 4. Glucose homeostasis in HFD mice overexpressing *Epgn* via adenovirus

(A) Schematic diagram of experiment, Adv-*Gfp* or Adv-*Epgn* was injected into mice fed with HFD for 12 weeks. (B) Relative *Epgn* mRNA levels in liver after euthanization. (C) Epigen levels increased in liver of adv-*Epgn* group. (D) Epigen levels in serum at day 3. (E) Body weight and (F) fasting glucose levels at various time points. (G) GTT results after 5 days injection of adenovirus. AUC (H) and GSIS (I) from the GTT in (G). PTT results (J) and corresponding AUC (K) after 7 days injection of adenovirus. ITT results (L) and AUC (M) after 10 days injection of adenovirus. $n = 6$ Adv-*Gfp*, 6 Adv-*Epgn*. Data are the mean ± SEM. Statistical analysis was performed using unpaired two-tailed Student's t test or Welch's t test. $^*p < 0.05$, $^{**}p < 0.01$.

Although epigen did not promote glucose uptake in the liver, the findings that *Epgn* knockout worsened glucose metabolism while *Epgn* overexpression and epigen treatment enhanced pyruvate tolerance (as demonstrated by the pyruvate tolerance test [PTT]) prompted us to explore whether epigen can inhibit liver utilization of pyruvate by repressing gluconeogenesis. Glucose production in primary hepatocytes infected with Adv-*Epgn* under glucagon and pyruvate induction was lower compared with hepatocytes infected with Adv-*Gfp* (Figure 5P). Treatment of primary mouse hepatocytes (Figure 5Q) or HepG2 cells (Figure S4L) with glucagon and pyruvate increased glucose levels in the cells via gluconeogenesis. However, treatment with recombinant epigen in primary hepatocytes (Figure 5Q) and HepG2 cells (Figure S4L) significantly lowered glucose levels. Taken together, epigen can directly promote glucose uptake by adipocytes and muscle cells and represses glucose production by hepatocytes.

Chronic injection of low-dosage epigen also alleviates metabolic dysregulation induced by HFD treatment

To assess the pharmaceutical potential of long-term epigen administration, we injected a low dose of recombinant epigen (1 mg/kg) into mice for 15 weeks (Figure 6A). Chronic epigen injection did not alter body weight (Figure 6B) but significantly reduced fat mass (Figures 6C and 6D). Both sWAT and eWAT weights were lower in the epigen-treated group compared with PBS controls, likely due to smaller adipocyte sizes in these tissues (Figures 6E–6G) and reduced immune cell infiltration (Figure 6H). Fasting glucose levels in the epigen-treated group were consistently lower than those in the control group starting at week 6 (Figure 6I). Additionally, chronic low-dose epigen injection improved glucose tolerance (Figures 6J–6M) and pyruvate tolerance (Figures 6N–6P), as shown by GTT and PTT, respectively. However, insulin sensitivity was not significantly enhanced (Figures 6Q and 6R).

Although there were no differences in VO_2 consumption (Figure S5A), VCO_2 production (Figure S5B), energy consumption (Figure S5C), locomotor activity (Figure S5F), and food intake (Figure S5E) between the groups, chronic injection of epigen significantly increased the respiratory exchange ratio (RER), particularly under lower temperature conditions (Figure S5D). While there were no differences in circulating TG and CHO levels (Figure S5G), chronic

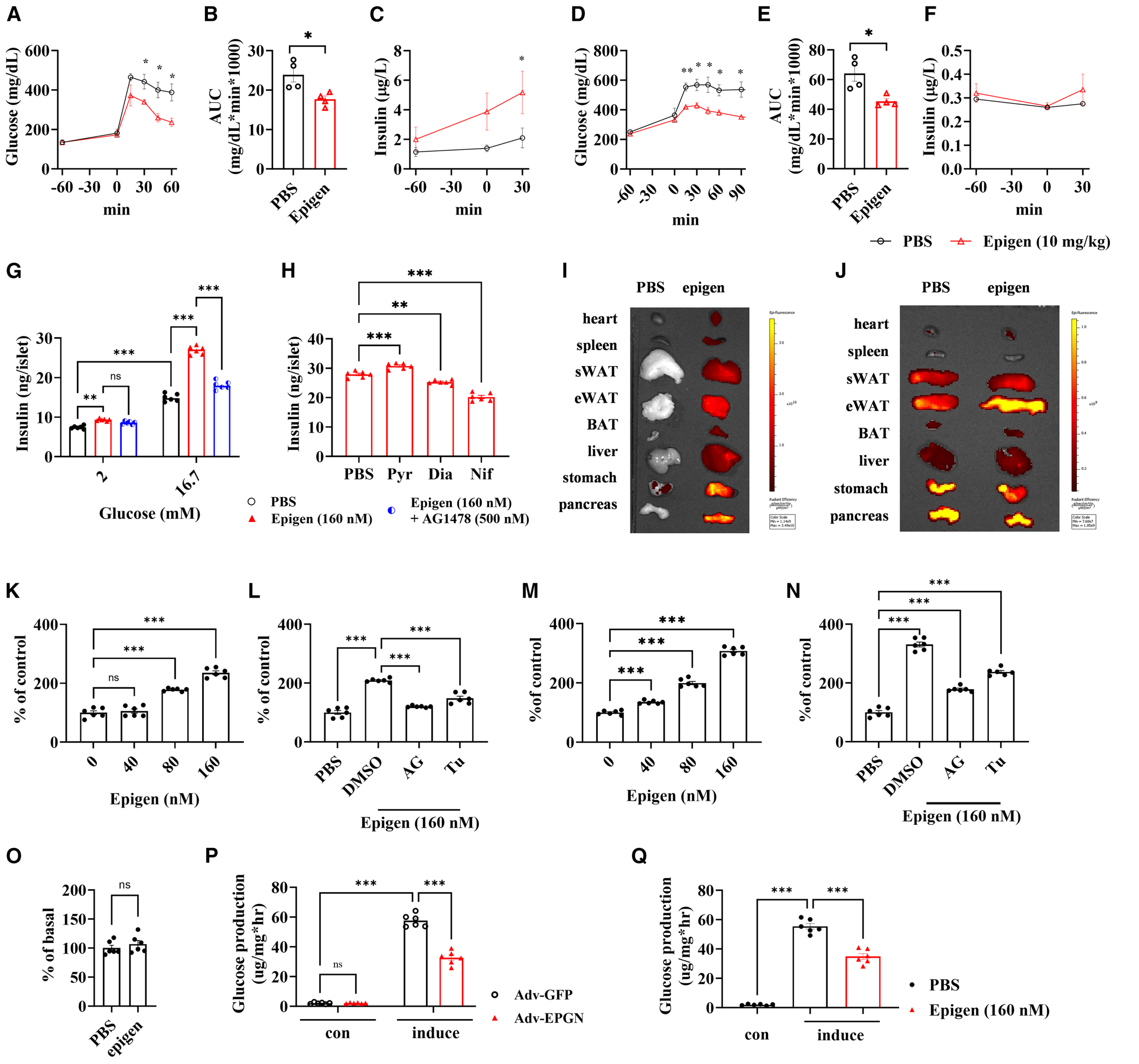

Figure 5. Glucose tolerance in mice following acute recombinant epigen treatment

(A) GTT results in 18-week HFD-fed mice. AUC (B) and GSIS (C) from the GTT in (A). (D) GTT results in STZ-treated mice. AUC (E) and GSIS (F) from the GTT in (D). (G and H) Epigen-induced insulin secretion in beta cells under high-glucose stimulation. (G) Insulin secretion in isolated mouse islets with and without EGF receptor inhibitor AG1478. (H) Insulin secretion in isolated mouse islets with and without inhibitors for insulin secretion. (I) IVIS image showing label-epigen protein distribution, and (J) IVIS image showing NBDG distribution in mice after injection of either PBS or label-epigen protein for an hour. (K) Glucose uptake in SVF-differentiated adipocytes with epigen treatment in a dose-dependent manner. (L) Glucose uptake in SVF-differentiated adipocytes with and without EGF receptor inhibitors. (M) Glucose uptake in primary myoblast-differentiated muscle cells with epigen treatment in a dose-dependent manner. (N) Glucose uptake in myoblast-differentiated muscle cells with and without EGFR inhibitors AG1478 and Tucatinib. (O) Glucose uptake in primary hepatocytes with epigen treatment. (P) Glucose production in primary hepatocytes under glucagon and pyruvate induction, with and without overexpression of EPGN via adenovirus. (Q) Gluconeogenesis in primary hepatocytes with epigen protein treatment. (A–F), $n = 4$. (G) and (H), $n = 6$. (K–Q), $n = 6$. Data are the mean ± SEM. Statistical analysis was performed using unpaired two-tailed Student's t test or Welch's t test. Group differences were determined via two-tailed analysis of variance (ANOVA) with Tukey's post hoc test. $^*p < 0.05$, $^{**}p < 0.01$, $^{***}p < 0.001$.

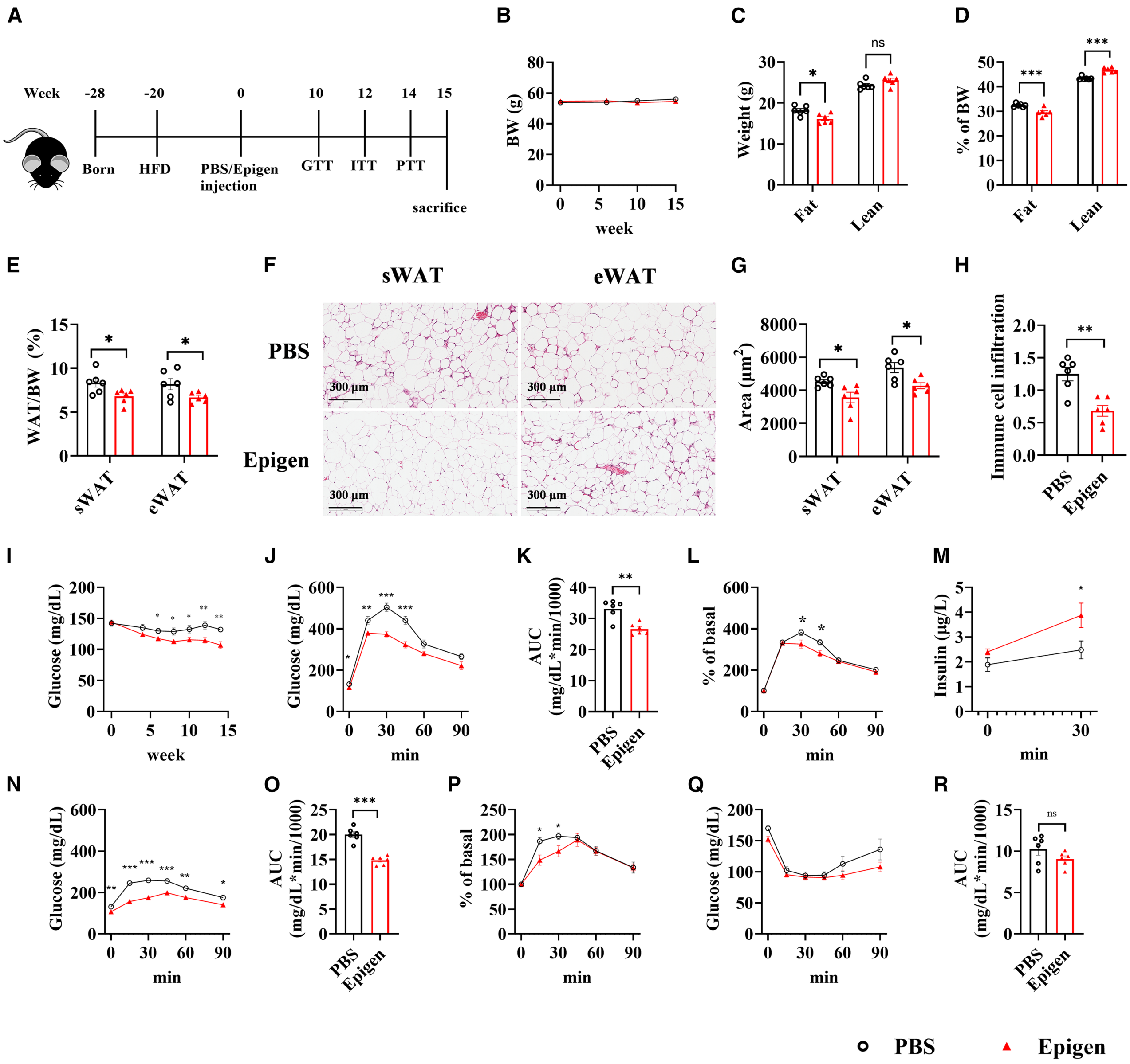

Figure 6. Fat mass and glucose homeostasis in HFD-induced obese mice following daily epigen injections

(A) Schematic diagram of experiment, recombinant epigen protein was intraperitoneally injected into mice fed with HFD for 20 weeks, and daily injection of epigen (2 mg/kg daily) for 15 weeks. (B) Body weight measured across various time points. (C) Fat and lean mass measurements. (D) Percentage of fat and lean mass relative to body weight. (E) Percentage of sWAT and eWAT mass relative to body weight. (F) Representative images of H&E staining of sWAT and eWAT, (G) quantification of adipocyte size of sWAT and eWAT, and (H) quantification of immune cell infiltration in sWAT at week 15. (I) Fast glucose levels across various time points. (J) GTT results at week 10. (K) AUC, (L) percentage of basal of GTT, and (M) insulin levels shown in (J). (N) PTT results at week 14, (O) AUC, and (P) percentage of basal of PTT shown in (N). (Q) ITT result and (R) AUC of the ITT in (Q) at week 12. $n = 6$. Data are the mean ± SEM. Statistical analysis was performed using unpaired two-tailed Student's t test or Welch's t test. $^{*}p < 0.05$, $^{**}p < 0.01$, $^{***}p < 0.001$.

injection of low-dose epigen lowered LDL levels and increased HDL levels (Figure S5H), as well as increased circulating FFA (Figure S5I) in HFD-fed mice. Hepatic TG level was decreased and no change in hepatic CHO level (Figure S5J). In agreement with lower hepatic TG level, lipid accumulation in the livers of mice receiving chronic epigen injection was lower than in the PBS control group, as demonstrated by H&E staining (Figures S5K and S5L). Less fibrosis and liver damage were also observed in the chronic epigen injection group, as shown by Sirius red staining (Figures S5M and S5N) and biochemical assays (Figure S5O).

Chronic injection of epigen reduces fat mass by enhancing respiration

Interestingly, increasing glucose uptake by adipocytes typically results in greater fat mass, as excess glucose is converted to lipids and stored in adipocytes.[29] However, to investigate the paradoxical reduction in fat mass observed in HFD-fed mice chronically injected with epigen, we performed RNA sequencing analysis on their sWAT. The mRNA profiles of sWAT from epigen-treated and control mice were significantly distinct, as shown by principal-component analysis (Figures S6A and S6B). Functional enrichment analysis revealed that epigen treatment affected biological processes related to respiration, cellular components involving mitochondrial proteins, and molecular functions involving GTP and ATP (Figure S6C). The heatmap in Figure S6D demonstrated that the expression of genes associated with the cellular respiration pathway in sWAT from HFD-fed mice injected with epigen was significantly higher than in the sWAT from their PBS controls. RT-qPCR was employed to validate these expression changes (Figure S6E).

To directly assess the impact of epigen on adipocyte respiration, we measured the oxygen consumption rate (OCR) and extracellular acidification rate (ECAR) in differentiated mouse 3T3-L1 cells and human SW872 cells using the Seahorse mito stress test and glycolytic rate assay. Notably, a 1-hour treatment with epigen enhanced both basal respiration, as indicated by OCR (Figures S6F and S6H), and glycolysis as demonstrated by ECAR (Figures S6G and S6I). These findings suggest that epigen promotes energy expenditure in adipocytes, which may explain the reduced fat mass despite increased glucose uptake.

Epigen promotes pancreatic beta cell regeneration in mice

As previous studies have shown that various EGFR ligands EGF[30,31] and TGF-α[32,33] can promote pancreatic beta cell regeneration, we investigated whether epigen could also facilitate this process. To evaluate the potential effects of epigen on pancreatic beta cell regeneration, we administered a low dose of epigen (2 mg/kg) via intraperitoneal injection to STZ-induced diabetic mice for 8 weeks (Figure 7A). Epigen treatment did not significantly affect body weight (Figure 7B) but reduced fasting glucose levels starting from week 4 (Figure 7C) and increased endogenous insulin levels from week 6 (Figure 7D). Consistent with the observations in HFD-fed mice (Figure 6), chronic epigen administration improved glucose tolerance, as evidenced by GTT results (Figures 7E–7G), and enhanced both basal and glucose-stimulated insulin secretion (Figure 7H). However, epigen did not improve insulin sensitivity, as shown by ITT results (Figures 7I and 7J). Furthermore, chronic epigen treatment slightly increased pancreatic mass (Figure 7K) and insulin content (Figure 7L). BrdU incorporation analysis revealed significantly higher beta cell proliferation in the epigen-treated group compared with PBS controls (Figures 7M and 7N). In summary, these findings indicate that chronic low-dose epigen administration enhances pancreatic beta cell regeneration and function, contributing to improved glucose tolerance, without affecting insulin sensitivity.

DISCUSSION

Foundational research on EGFR has revealed its critical role in cellular processes, including development and tissue repair. However, dysregulation of EGFR signaling has been strongly implicated in the pathogenesis of various diseases, particularly cancer.[2] Therefore, EGFR has been an important target for therapeutic interventions in cancer treatment. For example, EGFR inhibitors, including tyrosine kinase inhibitors (TKIs) and monoclonal antibodies, have been developed for clinical use to interfere with EGFR signaling and suppress the growth of cancer cells. However, it is worth noting that some of the EGFR TKIs, like rociletinib, have been associated with dose-limiting hyperglycemia. Patients receiving rociletinib treatment have reported the need for dose reduction, administration of oral antihyperglycemic agents, or both, to manage hyperglycemia while continuing therapy.[34] This suggests that EGFR signaling plays an important role in glucose homeostasis.

In this study, we demonstrated that circulating epigen levels are tightly regulated by nutritional status in mice and are correlated with BMI and HbA1c levels in humans. These findings suggest a potential role for circulating epigen level as a biomarker of metabolic health and glucose regulation. However, our cohort data included only random glucose levels, and it remains unclear whether fasting glucose levels influence circulating epigen levels. Future studies should address this limitation by evaluating the relationship between epigen and fasting glucose levels to provide deeper insights into its metabolic regulation. Albumin, a key marker of liver function and nutritional status, is often reduced in chronic inflammation or illness, while elevated globulin levels are associated with immune activity and inflammation. Despite these correlations, AST and ALT levels showed no significant differences, indicating that changes in epigen level are likely unrelated to liver injury and may instead reflect immune or inflammatory processes. Further studies are needed to explore the mechanistic role of epigen in inflammation and its potential as a biomarker for metabolic and immune-related diseases.

Nevertheless, our mouse models reveal that *Epgn* KO worsens glucose intolerance, likely due to impaired insulin secretion from pancreas and dysregulation of hepatic gluconeogenesis in DIO mice. In contrast, short-term overexpression of *Epgn* improves glucose and pyruvate tolerance in DIO mice while leaving body weight and insulin sensitivity unaffected. Future studies could explore longitudinal changes in epigen levels in subjects before and after the onset of diabetes, as well as investigate potential alterations in epigen levels in individuals with type 1 diabetes.

We also demonstrated that acute administration of recombinant epigen can lower blood glucose levels through both insulin-dependent and insulin-independent pathways. Mechanistically, epigen induces insulin secretion from pancreatic beta cells under high-glucose conditions via the activation of EGFR.[12,28] Our findings align with previous studies showing that EGFR activation stimulates insulin secretion through a concentration-dependent mechanism involving calcium influx, which can be inhibited by EGFR blockers.[12,28] Epigen

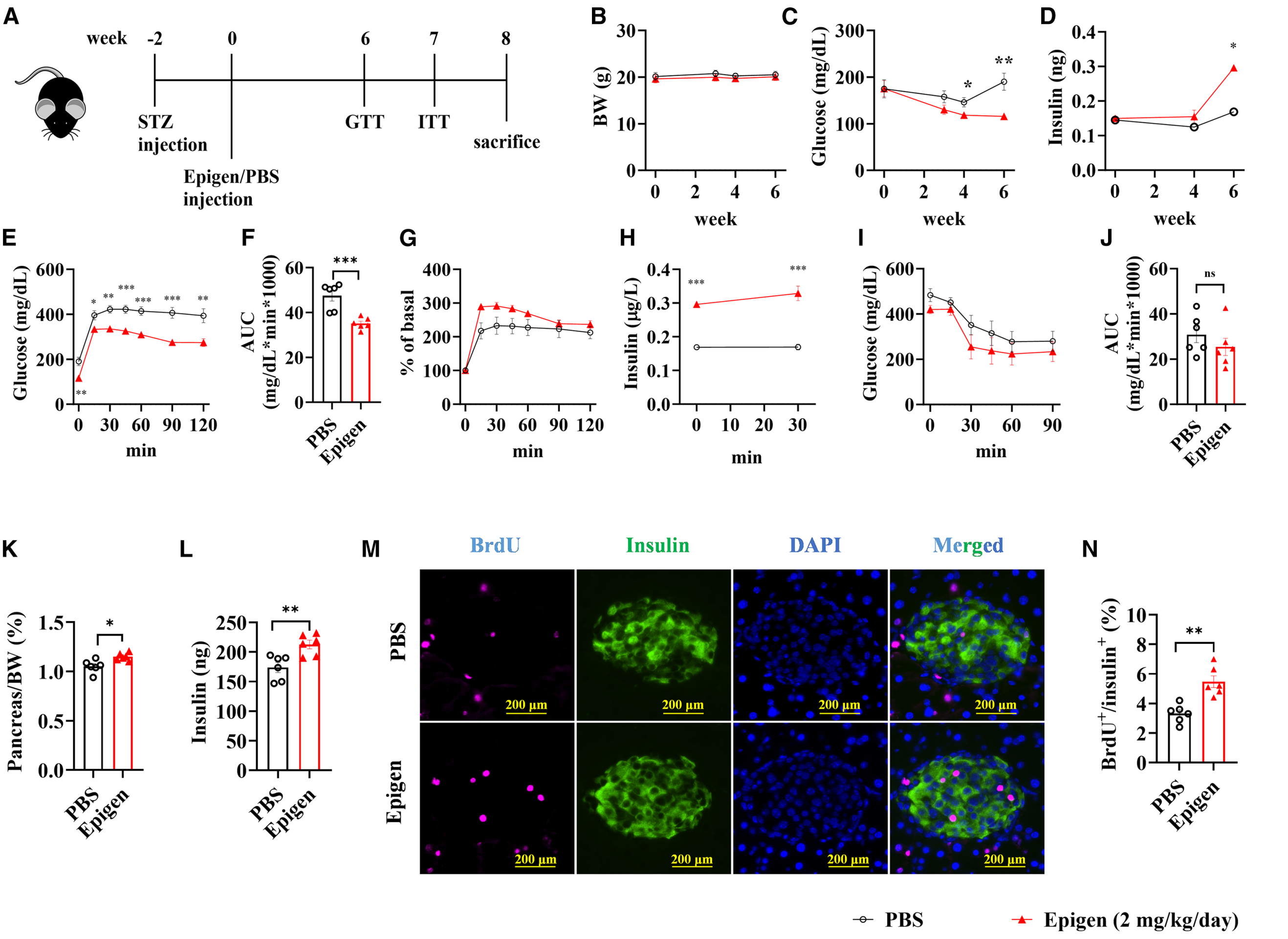

Figure 7. Glucose homeostasis in STZ-induced diabetic mice following daily epigen injections

(A) Schematic diagram of experiment. Body weight (B), fasting glucose (C), and fast insulin levels (D) at various time points. (E) GTT results at week 6. AUC (F), percentage of change (G), and GSIS (H) for the GTT shown at (E). ITT results (I) and the AUC (J) at week 7. Pancreas weight (K), total amount of insulin (L) in pancreas, representative images (M) of BrdU staining of pancreas, and quantification of BrdU+/insulin+ cells (N) at week 8. $n = 6$. Data are the mean ± SEM. Statistical analysis was performed using unpaired two-tailed Student's t test or Welch's t test. *$p < 0.05$, **$p < 0.01$, ***$p < 0.001$.

can also directly promote glucose uptake in adipocytes and muscle cells via the activation of EGFR signaling pathways.[3] Remarkably, while many interventions that increase insulin levels or sensitivity are widely used to improve glucose homeostasis, they often result in weight gain due to their effects on glucose utilization being redirected into fat storage.[29] This is in contrast to treatments like GLP-1 mimetics, which reduce food intake.[35] Although chronic treatment with epigen did not lower the total body weight, it significantly reduced fat mass by enhancing mitochondrial function in adipocytes, highlighting its potential direct role in improving metabolic health rather than indirectly through overall weight loss.

A previous study reported that EGF has been shown to enhance aerobic glycolysis in triple-negative breast cancer cells.[36] Importantly, we found that genes and pathways involved in respiration are upregulated in the epigen treatment groups, according to our RNA sequencing analysis. This observation leads us to hypothesize that there may be a novel pathway through which epigen enhances glycolysis as an energy source for adipocytes. This is indicated by the ECAR from the Seahorse mitochondrial stress test at the *in vitro* adipocyte level, as well as the increase in the RER observed in our metabolic cage study at the *in vivo* animal level. Current popular GLP-1 receptor agonists have gained attention for their efficacy in improving glycemic control and promoting weight loss. However, patients may experience muscle wasting as side effects.[37] These issues can significantly impact their overall well-being and quality of life. In contrast, our study found that epigen treatment did not lead to loss of lean mass, suggesting a more favorable outcome.

Indeed, a previous study reported that the glucose-lowering effect of intravenous EGF is mediated, at least in part, via insulin.[12] It was shown that a dose of 50 μg/kg EGF demonstrated a potency

comparable to 0.06 U/kg insulin in lowering glucose levels in db/db mice.[12] Furthermore, some studies have reported conflicting effects of EGF, including its ability to stimulate glycogenolysis in the liver and induce mild hyperglycemia in mice.[38] In contrast, higher dosages of epigen administered via intraperitoneal injection have been shown to result in a significant acute reduction in circulating glucose levels, as evidenced by GTTs, without causing notable hypoglycemia in both DIO and STZ diabetic mice. These findings suggest that epigen is likely a more promising candidate than EGF for further exploration as an anti-diabetic treatment. Further analysis is required to determine whether these differences arise from variations in their affinity for EGFR, and consequently, their glucose-lowering effect.

Although gene therapies for treating type 1 diabetes were proposed,[39] many hurdles have prevented their application in humans. In contrast, recombinant protein treatment still represents a more realistic approach. Previous studies have shown that twice-daily intraperitoneal injections of EGF (1 μg/kg) and gastrin (3 μg/kg) for 2 weeks restored normoglycemia after diabetes onset in NOD mice, whereas EGF or gastrin alone did not.[11] Previous studies have attempted to use EGFR ligands for diabetes treatment especially for the survival of pancreatic beta cells. Activation of EGFR has been shown to promote beta cell proliferation, differentiation, and survival, ultimately facilitating insulin production and release. The first *in vivo* study focusing on the early neonatal period of rats revealed that EGF treatment resulted in elevated plasma insulin concentrations and a higher proportion of beta cells within the islets of Langerhans in 1995.[40] Mechanistically, this improvement can be attributed to the regulation of survivin by EGF through the Raf-1/MEK/ERK pathway in pancreatic beta cells, where EGF prolongs the half-life of the survivin protein and inhibits its ubiquitin-mediated proteasomal degradation.[30] In addition to EGF, other EGFR ligands have also been reported to increase beta cell mass. For example, overexpression of TGF-α has been shown to induce the initiation of islet neogenesis via PDX1.[33] *In vitro*, betacellulin can convert acinar cells into insulin-producing cells[41]; however, in animal models, gene therapy using betacellulin and neurogenin 3 delivered via adenoviral vectors has reversed major metabolic problems in insulin-deficient diabetic mice.[42] Overexpressing HB-EGF also has a significant role in promoting beta cell proliferation via mTOR, MAPK, PI3K/AKT, and IRS2 pathways as demonstrated by the transgenic rat model.[14] However the evidence for epiregulin promotes the proliferation and secretory function limited to rat insulinoma cell lines.[43] While it remains unknown whether amphiregulin can promote beta cell proliferation, it appears to be a common feature among EGFR ligands. In this study, we showed that treatment of recombined epigen protein (2 mg/kg/d) can increase the endogenous insulin level and pancreatic beta cell mass in STZ-treated mice within 6 weeks. It remains to be explored whether this is a unique feature of epigen in a specific mouse model or just higher concentrations of EPGR ligands was used to promote beta cell regeneration and proliferation. More comprehensive experiments with detailed comparisons of each EPGR ligand across various preclinical type 1 diabetes mouse models and human beta cells are required.

EGFR ligands are proposed to treat various diseases, including metabolic disorders, but their involvement raises significant concerns regarding potential tumorigenesis. Nuclear-localized EGFR is closely associated with disease progression, resulting in worse overall survival in numerous cancers and enhanced resistance to radiation, chemotherapy, and anti-EGFR therapies such as gefitinib and cetuximab.[44] EGF, HB-EGF, and TGF-α are known to facilitate EGFR nuclear translocation, whereas amphiregulin and epiregulin do not promote this process.[45] While EGF, HB-EGF, and TGF-α exhibit high affinity for EGFR, amphiregulin and epiregulin have lower affinities, and the role of epigen in promoting nuclear localization of EGFR remains to be explored.[22] Therefore, the key questions remain regarding which EGFR ligands are most practical, potent, and safe for use in humans.

Previous studies involving EPGN transgenic mice, specifically those overexpressing EPGN in the skin, observed peripheral demyelination and sebaceous gland hyperplasia.[26] In this study, we did not observe any tumorigenesis in mice injected with adenovirus overexpressing EPGN or through chronic injection of epigen. Moreover, the expression of cancer marker mRNAs in the RNA sequencing data from livers and WATs remained unchanged (data not shown). This indicates that EPGN and epigen treatment do not induce tumorigenic effects, supporting their potential safety for therapeutic use., However, EGFR ligand can induce tumorigenesis by various mechanisms. Further investigation into the chronic treatment of epigen is required.

Limitations of the study

While this study provides compelling evidence for the role of epigen in glucose homeostasis and its potential as a therapeutic target for diabetes, several limitations should be acknowledged. First, the findings are predominantly based on mouse models, which may not fully capture the complexity of human physiology. Validation in human models, including cohorts of patients with type 2 and type 1 diabetes, is essential to confirm the translational relevance of our results, particularly regarding the effects of epigen on the proliferation and viability of human beta cells. Second, although epigen treatment demonstrated promising effects without inducing tumorigenesis in our experiments and others, long-term safety studies in diverse experimental systems are needed to rule out potential adverse effects, including tumorigenic risks.

Conclusions

This study highlights the multifaceted potential of epigen in glucose regulation and diabetes treatment. By demonstrating its ability to enhance insulin secretion, promote glucose uptake, and improve beta cell mass without inducing adverse effects such as weight loss or tumorigenesis, epigen emerges as a promising therapeutic candidate for both type 2 and type 1 diabetes. Its unique mechanism of action, distinct from other EGFR ligands, underscores the need for further research to fully explore its therapeutic applications and safety profile. These findings pave the way for the development of

epigen-based treatments that could offer effective and safe solutions for diabetes management.

MATERIALS AND METHODS

Animal experiments

All animal experiments were approved by Department of Health HKSAR Government (Ordinance Cap. 340), and the Animal Subjects Ethics Sub-Committee (ASESC) of the Hong Kong Polytechnic University. C57BL/6J WT mice were housed in the Centralized Animal Facilities of the Hong Kong Polytechnic University at 23°C ± 1°C on standard 12 h light/12 h dark cycle with ad libitum access to drinking water and diet of standard chow (STC) or high-fat diet (HFD).[27,46] For adenovirus-mediated *Epgn* overexpression, 10^9 PFU adenovirus expressing GFP (Adv-*Gfp*) or epigen (Adv-*Epgn*) was injected into mice intravenously. The STZ-induced diabetic mouse model was established as described in a previous report.[47]

Human samples

Human blood samples used in the study were selected with and without type 2 diabetes and obtained from the Chinese University of Hong Kong and Foshan Sanshui District People's Hospital. Informed consent has been obtained for research (approval by the Joint Chinese University of Hong Kong-New Territories East Cluster Clinical Research Ethics; Ref #2018.109 and Foshan Sanshui District People's Hospital; Ref#ZSY-KY-2025006). The serums were isolated from the blood sample and immediately snap-frozen in liquid nitrogen and stored at −80°C for further protein quantification analysis as described previously.[48]

Production of the recombinant active form of epigen

The backbone plasmid pLJSRSF7 was used to construct the expression plasmid pLJSRSF7-*Epgn*. The coding sequences of the active form of *Epgn* were amplified by PCR using synthetic oligonucleotides and inserted into vector with digestion and ligation with specific restriction enzymes. The constructed plasmid pLJSRSF7-*Epgn* was then transformed into BL21. The protein expression was induced with 100 μM Isopropyl β-d-1-thiogalactopyranoside. The bacterial cells were collected, and the target protein was purified with immobilized metal affinity chromatography (MaestroClin Limited).

Indirect calorimetry and body composition measurements

The mice were housed individually in metabolic cage system (8-channel, Promethion, Sable Systems) was used to monitor indirect calorimetry of mice as described previously.[49] The mice were fed for indicated days and temperature with free food and water. The metabolic and behavioral data were recorded and integrated by the MacroInterpreter analysis software. Body composition (fat mass and lean mass) was measured using Nuclear Magnetic Resonance (The Bruker minispec LF90II Body Composition Analyzer).

Ex vivo IVIS imaging of epigen and glucose delivery and distribution

DIO mice fed with HFD for 16 weeks were used in experiments. The epigen protein was labeled with fluorescent dye Alexa Fluor 647 with the Fluorescent Protein Labeling Kit (A20173, Invitrogen), following the manufacturer's instructions. Either fluorescent labeled protein or 200 mg/kg 2-deoxy-2-[(7-nitro-2,1,3-benzoxadiazol-4-yl) amino]-D-glucose (2-NBDG) was intraperitoneally injected. After an hour, the organs were collected and imaged with In Vivo Animal Imaging System (IVIS, PerkinElmer IVIS Lumina Series III).

Glucose, insulin, and pyruvate tolerance test

Glucose, insulin and pyruvate tolerance tests were performed in mice fasted overnight or after a 6-h fast, following which intraperitoneal injection of glucose (Bio Basic, 2 g/kg for STC, 1 g/kg for HFD and 0.5 g/kg BW for STZ mice) or insulin (Novo Nordisk, 0.5 U/kg for STC, 0.75 U/kg for HFD and 1.5 U/kg BW for STZ mice) or sodium pyruvate (Sigma, 1.5 g/kg for STC and 1 g/kg BW for HFD mice) was administered. Blood glucose measurements were assessed via tail whole blood at time point 0 (at the end of the fast and before glucose loading), 15-, 30-, 60- and 90-min post-glucose or insulin administration, using the glucose meter (Accu-chek).

Glucose-stimulated insulin secretion

For glucose-stimulated insulin secretion (GSIS) in mice, the serum separated from blood collected from the tail vein at −60, 0, and 30 min in GTT was subjected to measurement of insulin using a mouse insulin ELISA kit (10-1247-01, Mercodia). SJ beta cells and isolated islets were prepared for analysis by washing in secretion buffer (137 mM NaCl, 0.9 mM $CaCl_2$, 2.7 mM KCl, 1.5 mM KH_2PO_4, 0.5 mM $MgCl_2$, 8.1 mM Na_2HPO_4, 20 mM HEPES pH 7.4, 0.2% BSA), incubation in secretion buffer containing 2 mM glucose for 30 min, and again washing in secretion buffer. The assay was then performed by incubation for 30 min in secretion buffer containing indicated concentrations of glucose and secretagogues. The insulin level was determined using a mouse insulin ELISA kit (32100, ImmunoDiagnostics).

Mouse serum chemicals test

The serum was collected from heart after animals were euthanized or tail vein puncture. Serum chemicals or enzymes were measured using assay kits : total triglyceride (TG, 100000220, BioSino), total cholesterol (CHO, 100000180, BioSino), alanine aminotransferase (ALT, 2930-500, Stanbio), aspartate aminotransferase (AST, 2920-500, Stanbio), low-density-lipoprotein-cholesterol (LDL-C, A113-1-1, Nanjing Jiancheng), and high-density-lipoprotein-cholesterol (HDL-C, A112-2-1, Nanjing Jiancheng) .

Epigen level measurement

For mouse serum samples, ELISA wells were coated with 0.8 μg/mL goat polyclonal epigen antibody (AF1127, R&D Systems) in coating buffer at 4°C overnight, the wells were washed with TBST followed by blocking with 1% BSA for 1 h. Each well was added with 10 μL mouse serum and 90 μL blocking buffer, incubated in room

temperature for 2 h. The wells were washed with TBST followed by incubation with 0.5 μg/mL rabbit polyclonal epigen antibody (PA5-79208, Thermo Fisher) in blocking buffer for 2 h. The wells were washed with TBST followed by incubation with horseradish peroxidase (HRP)-rabbit antibody in blocking buffer for 1 h. The wells were washed with TBST followed by developing in TMB for 15 min and stopping with 2 M sulfuric acid. The signals were obtained by measuring OD value at 450 nm. For human serum samples, serum was diluted with coating buffer and added to ELISA wells and incubated for 2 h; the wells were washed with TBST followed by blocking with 1% BSA for 1 h. The wells were washed with TBST followed by incubation with 0.5 μg/mL epigen antibody (ab219978, Abcam) in blocking buffer for 2 h. The wells were washed with TBST followed by incubation with HRP-rabbit antibody in blocking buffer for 1 h. The wells were washed with TBST followed by developing in TMB for 15 min and stopping with 2 M sulfuric acid. The signals were obtained by measuring OD value at 450 nm.

Cell culture

Mouse 3T3-L1, human SW872, human HepG2, human A204, and rat L6 cell lines were grown and maintained in high-glucose DMEM containing 10% FBS and 1% antibiotic-antimycotic mixture in an atmosphere of 5% CO_2 at 37°C. Mouse SJ beta cells, an immortalized pancreatic beta cell line,[50] (Radvanyi et al., 1993), were grown and maintained in the same condition with an additional 55 μM 2-Mercaptoethanol. Isolated SVF, 3T3-L1, and SW872 cells were differentiated with MDI induction medium (high-glucose DMEM with 50 mM 3-isobutyl-1-methylxanthine [IBMX, Cayman], 1 mM dexamethasone [Cayman], and 10 μg/mL insulin), insulin medium (high-glucose DMEM with 1 μg/mL insulin), and high-glucose DMEM. L6 and A204 cells were differentiated by high-glucose DMEM containing 2% horse serum (OXOID).

Primary cell isolation and culture

The islets from mice were isolated as described in a previous report.[51] The stromal vascular fraction (SVF) from mouse adipose tissue was isolated following the protocol outlined in a previous report.[52] Primary muscle cells were differentiated from isolated myoblasts according to the method described in a previous report.[53] Primary hepatocytes from mice were isolated and cultured according to the procedure detailed in a previous report.[54]

Glucose production assay

Primary hepatocytes and HepG2 cells were washed with Hank's Balanced Salt Solution (HBSS, 127 mM NaCl, 3.5 mM KCl, 0.44 mM KH_2PO_4, 4.2 mM $NaHCO_3$, 0.33 mM Na_2HPO_4, 1 mM $CaCl_2$, and 20 mM HEPES, pH 7.4) for three times and starved in HBSS for 2 h, then washed again. The cells were incubated for 3 hours in HBSS buffer containing indicated reagents. After 3 h of incubation, the culture buffer was collected and centrifuged. The concentration of glucose in the supernatant was measured with the glucose content assay kit (Catalog #BC2505, Solarbio Science and Technology) according to the manufacturer's instructions.

Glucose uptake assay

The differentiated cells were fasted in serum-free high-glucose DMEM overnight, followed by starvation in glucose-free DMEM containing 1% fatty acid-free BSA (Solabio) for 3 h. The starved cells were incubated with indicated compounds for various times before glucose uptake assay start. The glucose uptake assay was performed using the glucose uptake kit following the manufacturer's instructions (ab136955, Abcam).

Real-time PCR

Total RNA was extracted from mice tissues with TRIzol and 2.5–5 μg of total RNA was used for cDNA synthesis by reverse transcription reaction. The expression level of target genes was detected using SYBR Green (Q411-02, Vazyme) with the specific primers on a Real-Time PCR System (Roche). Housekeeping genes were used for normalization.

Immunoblotting

Protein in cells or tissues was extracted with RIPA lysis buffer and protein concentration was determined by BCA protein quantification assay kit (PC0020, Solarbio). Extracted proteins were denatured by sample loading buffer under 95°C for 5 min. Equal proteins were loaded into and separated by sodium dodecyl sulfate-polyacrylamide gel electrophoresis (SDS-PAGE), and then transferred to PVDF membrane with constant 100 voltage for 90 min. After blocking with 5% non-fat milk in 1 X TBST for 1 h at room temperature, the membrane was probed with different primary antibodies in 5% BSA in 1 X TBST at 4°C for overnight. The membrane was washed with 1 X TBST three times (5 min each) and subsequently incubated with HRP-conjugated secondary antibodies prepared in 5% non-fat milk in 1 X TBST for 1 h at room temperature. Afterward, the membrane was washed with 1 X TBST for four times (10 min each). The specific signal of protein was visualized by ECL western blot detection kit (PE0010, Solarbio). The intensity of protein bands was quantified by ImageJ software, relative target protein expression levels were normalized by indicated internal control.

Hepatic lipids

Approximately 25 mg of liver tissues were homogenized with 200 μL PBS, 10 μL of the homogenate was subjected to protein quantification, and the rest of the homogenate was followed by mixing with 5 mL of a chloroform-methanol mixture (chloroform:methanol, 2:1, v/v) at 4°C overnight. An aliquot of 1 mL of the mixture was washed with 200 μL of saline, followed by centrifugation at 5,000 × *g* for 15 min and the washing procedure was repeated. The chloroform layer was collected and air-dried, and then resuspended in 100% ethanol. The TG levels were measured and normalized with protein concentration of tissue lysates.

Histology

After mice were euthanized, tissues were placed into tissue embedding cassettes and fixed in 10% neutral formalin for 24 h. Tissues were then processed with an Excelsior AS Tissue Processor using the following protocol: 30 min in 75% ethanol; 75 min

in 95% ethanol × 2 times; 75 min in 95% ethanol × 3 times; 60 min in xylene × 2 times; overnight paraffin infiltration at 60°C. Tissues were subsequently embedded in freshly melted paraffin and stored until further analysis. The paraffin-embedded blocks were cut into 5-μm-thick sections, which were deparaffinized and rehydrated by 15 min xylene × 2 times, 5 min 100% ethanol × 2 times, 1 min 95% ethanol × 2 times, 1 min 70% ethanol × once and then rinsed in tap water, then the slides were ready for following staining.

Hematoxylin and eosin staining

For hematoxylin and eosin (H&E) staining, the sections were stained with hematoxylin for 30 s for liver or brown adipose tissue and 3 min for white adipose tissue. Then the sections were stained with eosin for 45 s for liver or brown adipose tissue and 10 min for white adipose tissue, followed by rinse in tap water and dehydration (1 min 70% ethanol once, 1 min 95% ethanol for two times, 5 min 100% ethanol for two times). Finally, the stained sections were immersed in xylene and then mounted with DPX mounting medium.

Sirius red staining

Collagen I and III fibers were stained by Sirius red to evaluate fibrosis stage in tissue. Sections were immersed in Picro Sirius red solution and stain for 60 min at room temperature, then rinsed quickly in acetic acid solution, followed by absolute alcohol, followed by rinse in tap water and dehydration. Finally, the stained sections were mounted with DPX mounting medium.

RNA-seq analysis

Total RNA in fresh mice tissue was extracted using TRIzol reagent and cleaned up by RNeasy Mini Kit in accordance with the instructions as described previously.[46] The RNA quality was determined using RNA 6000 Pico kit by Agilent 2100 Bioanalyzer. After the quality check, mRNA was purified from total RNA using poly-T oligo-attached magnetic beads according to the manufacturer's instructions of Guangzhou Angte Biotech Co., Ltd (Guangzhou, China). The sequencing of all RNA samples was performed using DNBSEQ platform via 100-base pair reads from the paired ends. With raw sequencing data, simple quality control metrics were generated using fastQC. The reads were aligned to the mouse genome (Genome assembly: GRCm39) using STAR. Raw reads counts were quantified using the feature Counts function of the Rsubread package. Differential expression genes (DEGs) analysis was performed using the DESeq2 R package, which internally corrected for the library size. GO functional enrichment analysis, which included biological process (BP), molecular function (MF), and cellular component (CC), were performed using the clusterProfiler R package. Adjusted $p < 0.05$ was considered statistically significant.

Total insulin content determination

The pancreas was dissected, weighed, and homolyzed in 5 mL of acid-ethanol (2% concentrated HCL in 100% ethanol), supernatant was isolated after centrifuge at 4°C 4,000 rpm for 5 min. The insulin content was measured in the supernatant with insulin kit (Mercodia).

Bioenergetic analysis

The Seahorse XF24 Analyzer instrument (Agilent Seahorse Bioscience, CA, USA) and the related consumables (plates, cartridges, and inhibitor kits) were used according to the manufacturer's instructions. The 3T3-L1 and SW872 cells were seeded and differentiated in Seahorse 24-well XF Cell Culture Plate. The differentiated 3T3-L1 and SW872 cells were pretreated with PBS/160 nM epigen protein 1 h before assay. For the extracellular acidification rate (ECAR) determination, the cells were analyzed using the Seahorse XF glycolysis stress test kit. After baseline measurement, the following injections were made: 10 mM glucose, 1 μM oligomycin, and 50 mM 2-deoxyglucose (2-DG). For the oxygen consumption rate (OCR) determination, the cells were analyzed using the Seahorse XF Cell Mito stress test kit. After baseline measurement, the following injections were made: 1 μM oligomycin, 1 μM carbonyl cyanide-4 (trifluoromethoxy) phenylhydrazone (FCCP), and 1 μM rotenone/antimycin A (AA).

Statistical analysis

Figures were generated by GraphPad Prism 10. Histological images were quantified with ImageJ. Significance was determined using 2-sided Student's t test with Bonferroni correction or repeated-measures one-way ANOVA with Bonferroni correction except specific mention. A p value of less than 0.05 represented statistical significance. Correlations and differential analysis of human samples were analyzed with IBM SPSS Statistics 27. Significance was determined using Spearman's rho.

DATA AVAILABILITY

All data presented in the main text or the supplemental information are available from the corresponding author upon request.

ACKNOWLEDGMENTS

This work was supported by internal grants from The Hong Kong Polytechnic University and a grant from The Hong Kong University Grants Committee (AoE/M-707/18) to C.-M.W.

AUTHOR CONTRIBUTIONS

Conceptualization, K.-Y.C. and C.-M.W.; methodology and investigations, K.-Y.C., C.-J. D., T.-H.L., and C.-M.W.; study design, K.-Y.C. and C.-M.W.; resources, D.C. and P.P.Y. L.; writing – original draft, K.-Y.C. and C.-M.W.; validation, S.J. and P.P.Y.L.; project administration, C.-M.W.; funding acquisition, C.-M.W. All authors revised and approved the manuscript.

DECLARATION OF INTERESTS

The Hong Kong Polytechnic University filled the patent for epigen, with K.-Y.C., and C.-M.W. listed as the inventors.

DECLARATION OF GENERATIVE AI AND AI-ASSISTED TECHNOLOGIES IN THE WRITING PROCESS

During the preparation of this work the authors used GenAI to check grammar and syntax of selected sentences. After using this tool, the authors reviewed and edited the content as needed and take full responsibility for the content of the publication.

SUPPLEMENTAL INFORMATION

Supplemental information can be found online at https://doi.org/10.1016/j.ymthe.2025.06.044.

REFERENCES

1. Zhang, C., Jin, Y., Marchetti, M., Lewis, M.R., Hammouda, O.T., and Edgar, B.A. (2022). EGFR signaling activates intestinal stem cells by promoting mitochondrial biogenesis and beta-oxidation. Curr. Biol. *32*, 3704–3719.e7. https://doi.org/10.1016/j.cub.2022.07.003.
2. Sigismund, S., Avanzato, D., and Lanzetti, L. (2018). Emerging functions of the EGFR in cancer. Mol. Oncol. *12*, 3–20. https://doi.org/10.1002/1878-0261.12155.
3. Orofiamma, L.A., Vural, D., and Antonescu, C.N. (2022). Control of cell metabolism by the epidermal growth factor receptor. Biochim. Biophys. Acta Mol. Cell Res. *1869*, 119359. https://doi.org/10.1016/j.bbamcr.2022.119359.
4. Beytagh, M.C., and Weiss, W.A. (2022). EGFR ligands dictate tumour suppression. Nat. Cell Biol. *24*, 1189–1191. https://doi.org/10.1038/s41556-022-00967-z.
5. Strachan, L., Murison, J.G., Prestidge, R.L., Sleeman, M.A., Watson, J.D., and Kumble, K.D. (2001). Cloning and biological activity of epigen, a novel member of the epidermal growth factor superfamily. J. Biol. Chem. *276*, 18265–18271. https://doi.org/10.1074/jbc.M006935200.
6. Kwon, I.G., Kang, C.W., Park, J.P., Oh, J.H., Wang, E.K., Kim, T.Y., Sung, J.S., Park, N., Lee, Y.J., Sung, H.J., et al. (2021). Serum glucose excretion after Roux-en-Y gastric bypass: a potential target for diabetes treatment. Gut *70*, 1847–1856. https://doi.org/10.1136/gutjnl-2020-321402.
7. Oh, Y.S., Shin, S., Li, H.Y., Park, E.Y., Lee, S.M., Choi, C.S., Lim, Y., Jung, H.S., and Jun, H.S. (2015). Betacellulin ameliorates hyperglycemia in obese diabetic db/db mice. J. Mol. Med. *93*, 1235–1245. https://doi.org/10.1007/s00109-015-1303-1.
8. Song, M.Y., Bae, U.J., Jang, K.Y., and Park, B.H. (2014). Transplantation of betacellulin-transduced islets improves glucose intolerance in diabetic mice. Exp. Mol. Med. *46*, e98. https://doi.org/10.1038/emm.2014.24.
9. Li, L., Seno, M., Yamada, H., and Kojima, I. (2003). Betacellulin improves glucose metabolism by promoting conversion of intraislet precursor cells to beta-cells in streptozotocin-treated mice. Am. J. Physiol. Endocrinol. Metab. *285*, E577–E583. https://doi.org/10.1152/ajpendo.00120.2003.
10. Kristensen, K., Pedersen, S.B., Vinter-Jensen, L., Flyvbjerg, A., and Richelsen, B. (1998). Systemic administration of epidermal growth factor reduces fat mass in rats: effects on the hormone-sensitive-lipase, lipoprotein lipase and leptin. Horm. Res. *50*, 292–296. https://doi.org/10.1159/000023293.
11. Suarez-Pinzon, W.L., Yan, Y., Power, R., Brand, S.J., and Rabinovitch, A. (2005). Combination therapy with epidermal growth factor and gastrin increases beta-cell mass and reverses hyperglycemia in diabetic NOD mice. Diabetes *54*, 2596–2601. https://doi.org/10.2337/diabetes.54.9.2596.
12. Lee, H.Y., Yea, K., Kim, J., Lee, B.D., Chae, Y.C., Kim, H.S., Lee, D.W., Kim, S.H., Cho, J.H., Jin, C.J., et al. (2008). Epidermal growth factor increases insulin secretion and lowers blood glucose in diabetic mice. J. Cell. Mol. Med. *12*, 1593–1604. https://doi.org/10.1111/j.1582-4934.2007.00169.x.
13. Yasmeen, R., Shen, Q., Lee, A., Leung, J.H., Kowdley, D., DiSilvestro, D.J., Xu, L., Yang, K., Maiseyeu, A., Bal, N.C., et al. (2018). Epiregulin induces leptin secretion and energy expenditure in high-fat diet-fed mice. J. Endocrinol. *239*, 377–388. https://doi.org/10.1530/JOE-18-0289.
14. Maachi, H., Fergusson, G., Ethier, M., Brill, G.N., Katz, L.S., Honig, L.B., Metukuri, M.R., Scott, D.K., Ghislain, J., and Poitout, V. (2020). HB-EGF Signaling Is Required for Glucose-Induced Pancreatic beta-Cell Proliferation in Rats. Diabetes *69*, 369–380. https://doi.org/10.2337/db19-0643.
15. Fukatsu, Y., Noguchi, T., Hosooka, T., Ogura, T., Kotani, K., Abe, T., Shibakusa, T., Inoue, K., Sakai, M., Tobimatsu, K., et al. (2009). Muscle-specific overexpression of heparin-binding epidermal growth factor-like growth factor increases peripheral glucose disposal and insulin sensitivity. Endocrinology *150*, 2683–2691. https://doi.org/10.1210/en.2008-1647.
16. Kochupurakkal, B.S., Harari, D., Di-Segni, A., Maik-Rachline, G., Lyass, L., Gur, G., Kerber, G., Citri, A., Lavi, S., Eilam, R., et al. (2005). Epigen, the last ligand of ErbB receptors, reveals intricate relationships between affinity and mitogenicity. J. Biol. Chem. *280*, 8503–8512. https://doi.org/10.1074/jbc.M413919200.
17. Sahin, U., and Blobel, C.P. (2007). Ectodomain shedding of the EGF-receptor ligand epigen is mediated by ADAM17. FEBS Lett. *581*, 41–44. https://doi.org/10.1016/j.febslet.2006.11.074.
18. Wrenn, E.D., Yamamoto, A., Moore, B.M., Huang, Y., McBirney, M., Thomas, A.J., Greenwood, E., Rabena, Y.F., Rahbar, H., Partridge, S.C., and Cheung, K.J. (2020). Regulation of Collective Metastasis by Nanolumenal Signaling. Cell *183*, 395–410.e19. https://doi.org/10.1016/j.cell.2020.08.045.
19. Taniguchi, K., Yamamoto, S., Aoki, S., Toda, S., Izuhara, K., and Hamasaki, Y. (2011). Epigen is induced during the interleukin-13-stimulated cell proliferation in murine primary airway epithelial cells. Exp. Lung Res. *37*, 461–470. https://doi.org/10.3109/01902148.2011.596894.
20. Nagai, K., Arai, H., Okudera, M., Yamamura, T., Oki, H., and Komiyama, K. (2014). Epiregulin is critical for the acinar cell regeneration of the submandibular gland in a mouse duct ligation model. J. Oral Pathol. Med. *43*, 378–387. https://doi.org/10.1111/jop.12145.
21. Wurster, S., Ruiz, O.E., Samms, K.M., Tatara, A.M., Albert, N.D., Kahan, P.H., Nguyen, A.T., Mikos, A.G., Kontoyiannis, D.P., and Eisenhoffer, G.T. (2021). EGF-mediated suppression of cell extrusion during mucosal damage attenuates opportunistic fungal invasion. Cell Rep. *34*, 108896. https://doi.org/10.1016/j.celrep.2021.108896.
22. Freed, D.M., Bessman, N.J., Kiyatkin, A., Salazar-Cavazos, E., Byrne, P.O., Moore, J.O., Valley, C.C., Ferguson, K.M., Leahy, D.J., Lidke, D.S., and Lemmon, M.A. (2017). EGFR Ligands Differentially Stabilize Receptor Dimers to Specify Signaling Kinetics. Cell *171*, 683–695.e18. https://doi.org/10.1016/j.cell.2017.09.017.
23. Schneider, M.R., and Yarden, Y. (2014). Structure and function of epigen, the last EGFR ligand. Semin. Cell Dev. Biol. *28*, 57–61. https://doi.org/10.1016/j.semcdb.2013.12.011.
24. Dahlhoff, M., Schäfer, M., Wolf, E., and Schneider, M.R. (2013). Genetic deletion of the EGFR ligand epigen does not affect mouse embryonic development and tissue homeostasis. Exp. Cell Res. *319*, 529–535. https://doi.org/10.1016/j.yexcr.2012.11.001.
25. Dahlhoff, M., Emrich, D., Wolf, E., and Schneider, M.R. (2013). Increased activation of the epidermal growth factor receptor in transgenic mice overexpressing epigen causes peripheral neuropathy. Biochim. Biophys. Acta *1832*, 2068–2076. https://doi.org/10.1016/j.bbadis.2013.07.011.
26. Dahlhoff, M., Frances, D., Kloepper, J.E., Paus, R., Schäfer, M., Niemann, C., and Schneider, M.R. (2014). Overexpression of epigen during embryonic development induces reversible, epidermal growth factor receptor-dependent sebaceous gland hyperplasia. Mol. Cell. Biol. *34*, 3086–3095. https://doi.org/10.1128/MCB.00302-14.
27. Lee, J.T.H., Huang, Z., Pan, K., Zhang, H.J., Woo, C.W., Xu, A., and Wong, C.M. (2016). Adipose-derived lipocalin 14 alleviates hyperglycaemia by suppressing both adipocyte glycerol efflux and hepatic gluconeogenesis in mice. Diabetologia *59*, 604–613. https://doi.org/10.1007/s00125-015-3813-3.
28. Ishii, T., Miyasato, Y., Ichijo, M., Uchimura, K., and Furuya, F. (2023). Membrane protease prostasin promotes insulin secretion by regulating the epidermal growth factor receptor pathway. Sci. Rep. *13*, 9086. https://doi.org/10.1038/s41598-023-36326-7.
29. Frikke-Schmidt, H., Pedersen, T.Å., Fledelius, C., Olsen, G.S., and Hellerstein, M. (2013). Adipose weight gain during chronic insulin treatment of mice results from changes in lipid storage without affecting de novo synthesis of palmitate. PLoS One *8*, e76060. https://doi.org/10.1371/journal.pone.0076060.
30. Wang, H., Gambosova, K., Cooper, Z.A., Holloway, M.P., Kassai, A., Izquierdo, D., Cleveland, K., Boney, C.M., and Altura, R.A. (2010). EGF regulates survivin stability through the Raf-1/ERK pathway in insulin-secreting pancreatic beta-cells. BMC Mol. Biol. *11*, 66. https://doi.org/10.1186/1471-2199-11-66.
31. Yang, L., Zhu, Y., Kong, D., Gong, J., Yu, W., Liang, Y., Nie, Y., and Teng, C.B. (2019). EGF suppresses the expression of miR-124a in pancreatic beta cell lines via ETS2 activation through the MEK and PI3K signaling pathways. Int. J. Biol. Sci. *15*, 2561–2575. https://doi.org/10.7150/ijbs.34985.
32. Roder, P.V., Wu, B., Liu, Y., and Han, W. (2016). Pancreatic regulation of glucose homeostasis. Exp. Mol. Med. *48*, e219. https://doi.org/10.1038/emm.2016.6.
33. Song, S.Y., Gannon, M., Washington, M.K., Scoggins, C.R., Meszoely, I.M., Goldenring, J.R., Marino, C.R., Sandgren, E.P., Coffey, R.J., Jr., Wright, C.V., and

Leach, S.D. (1999). Expansion of Pdx1-expressing pancreatic epithelium and islet neogenesis in transgenic mice overexpressing transforming growth factor alpha. Gastroenterology *117*, 1416–1426. https://doi.org/10.1016/s0016-5085(99)70292-1.

34. Villadolid, J., Ersek, J.L., Fong, M.K., Sirianno, L., and Story, E.S. (2015). Management of hyperglycemia from epidermal growth factor receptor (EGFR) tyrosine kinase inhibitors (TKIs) targeting T790M-mediated resistance. Transl. Lung Cancer Res. *4*, 576–583. https://doi.org/10.3978/j.issn.2218-6751.2015.10.01.

35. Zhang, Y., Zhao, X., Dong, X., Zhang, Y., Zou, H., Jin, Y., Guo, W., Zhai, P., Chen, X., and Kharitonenkov, A. (2023). Activity-balanced GLP-1/GDF15 dual agonist reduces body weight and metabolic disorder in mice and non-human primates. Cell Metab. *35*, 287–298.e4. https://doi.org/10.1016/j.cmet.2023.01.001.

36. Lim, S.O., Li, C.W., Xia, W., Lee, H.H., Chang, S.S., Shen, J., Hsu, J.L., Raftery, D., Djukovic, D., Gu, H., et al. (2016). EGFR Signaling Enhances Aerobic Glycolysis in Triple-Negative Breast Cancer Cells to Promote Tumor Growth and Immune Escape. Cancer Res. *76*, 1284–1296. https://doi.org/10.1158/0008-5472.CAN-15-2478.

37. Linge, J., Birkenfeld, A.L., and Neeland, I.J. (2024). Muscle Mass and Glucagon-Like Peptide-1 Receptor Agonists: Adaptive or Maladaptive Response to Weight Loss? Circulation *150*, 1288–1298. https://doi.org/10.1161/CIRCULATIONAHA.124.067676.

38. Grau, M., Tebar, F., RAMÍREZ, I., and Soley, M. (1996). Epidermal growth factor administration decreases liver glycogen and causes mild hyperglycaemia in mice. Biochem. J. *315*, 289–293. https://doi.org/10.1042/bj3150289.

39. D'Addio, F., Maestroni, A., Assi, E., Ben Nasr, M., Amabile, G., Usuelli, V., Loretelli, C., Bertuzzi, F., Antonioli, B., Cardarelli, F., et al. (2022). The IGFBP3/TMEM219 pathway regulates beta cell homeostasis. Nat. Commun. *13*, 684. https://doi.org/10.1038/s41467-022-28360-2.

40. Sato, Y., Hiramatsu, Y., and Kudo, T. (1995). Effects of epidermal growth factor on glucose metabolism in neonatal rats. Biol. Neonate *68*, 26–32. https://doi.org/10.1159/000244214.

41. Mashima, H., Ohnishi, H., Wakabayashi, K., Mine, T., Miyagawa, J., Hanafusa, T., Seno, M., Yamada, H., and Kojima, I. (1996). Betacellulin and activin A coordinately convert amylase-secreting pancreatic AR42J cells into insulin-secreting cells. J. Clin. Invest. *97*, 1647–1654. https://doi.org/10.1172/JCI118591.

42. Yechoor, V., Liu, V., Paul, A., Lee, J., Buras, E., Ozer, K., Samson, S., and Chan, L. (2009). Gene therapy with neurogenin 3 and betacellulin reverses major metabolic problems in insulin-deficient diabetic mice. Endocrinology *150*, 4863–4873. https://doi.org/10.1210/en.2009-0527.

43. Kuntz, E., Broca, C., Komurasaki, T., Kaltenbacher, M.C., Gross, R., Pinget, M., and Damgé, C. (2005). Effect of epiregulin on pancreatic beta cell growth and insulin secretion. Growth Factors *23*, 285–293. https://doi.org/10.1080/08977190500233367.

44. Brand, T.M., Iida, M., Luthar, N., Starr, M.M., Huppert, E.J., and Wheeler, D.L. (2013). Nuclear EGFR as a molecular target in cancer. Radiother. Oncol. *108*, 370–377. https://doi.org/10.1016/j.radonc.2013.06.010.

45. Faria, J.A.Q.A., de Andrade, C., Goes, A.M., Rodrigues, M.A., and Gomes, D.A. (2016). Effects of different ligands on epidermal growth factor receptor (EGFR) nuclear translocation. Biochem. Biophys. Res. Commun. *478*, 39–45. https://doi.org/10.1016/j.bbrc.2016.07.097.

46. Wu, M., Lo, T.-H., Li, L., Sun, J., Deng, C., Chan, K.-Y., Li, X., Yeh, S.T.-Y., Lee, J.T. H., Lui, P.P.Y., et al. (2023). Amelioration of non-alcoholic fatty liver disease by targeting adhesion G protein-coupled receptor F1 (Adgrf1). Elife *12*, e85131.

47. Furman, B.L. (2015). Streptozotocin-Induced Diabetic Models in Mice and Rats. Curr. Protoc. Pharmacol. *70*, 5.47.1–5.47.20. https://doi.org/10.1002/0471141755.ph0547s70.

48. Ye, D., Li, H., Wang, Y., Jia, W., Zhou, J., Fan, J., Man, K., Lo, C., Wong, C., Wang, Y., et al. (2016). Circulating Fibroblast Growth Factor 21 Is A Sensitive Biomarker for Severe Ischemia/reperfusion Injury in Patients with Liver Transplantation. Sci. Rep. *6*, 19776. https://doi.org/10.1038/srep19776.

49. Wong, C.M., Wang, Y., Lee, J.T.H., Huang, Z., Wu, D., Xu, A., and Lam, K.S.L. (2014). Adropin is a brain membrane-bound protein regulating physical activity via the NB-3/Notch signaling pathway in mice. J. Biol. Chem. *289*, 25976–25986. https://doi.org/10.1074/jbc.M114.576058.

50. Radvanyi, F., Christgau, S., Baekkeskov, S., Jolicoeur, C., and Hanahan, D. (1993). Pancreatic beta cells cultured from individual preneoplastic foci in a multistage tumorigenesis pathway: a potentially general technique for isolating physiologically representative cell lines. Mol. Cell. Biol. *13*, 4223–4232.

51. Li, D.S., Yuan, Y.H., Tu, H.J., Liang, Q.L., and Dai, L.J. (2009). A protocol for islet isolation from mouse pancreas. Nat. Protoc. *4*, 1649–1652. https://doi.org/10.1038/nprot.2009.150.

52. Kilroy, G., Dietrich, M., Wu, X., Gimble, J.M., and Floyd, Z.E. (2018). Isolation of Murine Adipose-Derived Stromal/Stem Cells for Adipogenic Differentiation or Flow Cytometry-Based Analysis. Methods Mol. Biol. *1773*, 137–146. https://doi.org/10.1007/978-1-4939-7799-4_11.

53. Kim, K.H., Qiu, J., and Kuang, S. (2020). Isolation, Culture, and Differentiation of Primary Myoblasts Derived from Muscle Satellite Cells. Bio. Protoc. *10*, e3686. https://doi.org/10.21769/BioProtoc.3686.

54. Jung, Y., Zhao, M., and Svensson, K.J. (2020). Isolation, culture, and functional analysis of hepatocytes from mice with fatty liver disease. STAR Protoc. *1*, 100222. https://doi.org/10.1016/j.xpro.2020.100222.

YMTHE, Volume *33*

Supplemental Information

Epigen-mediated mechanisms to alleviate glucose homeostasis disruptions in diet-induced obese and STZ-induced diabetic mice

Ka-Ying Chan, Chu-Jun Deng, Dilun Chen, Tak-Ho Lo, Shiqi Jia, Pauline Po Yee Lui, and Chi-Ming Wong

Table S1 Baseline characteristics of study cohorts of normal or overweight/obese individuals. Data represent as mean ± SEM. Abbreviations: HbA1c, glycated haemoglobin, BMI, body mass index, SP, systolic pressures, DP, diastolic pressure, A/G, ALB/GLB ratio, ALB, albumin, GLB, globulin, ALP, alkaline phosphatase, ALT, alanine aminotransferase, AST, aspartate aminotransferase, GGT, Gamma-glutamyl transferase, DBIL, direct bilirubin, IBIL, indirect bilirubin, TBIL, total bilirubin.

	Normal	Overweight/Obese
Gender M/F	37/36	39/11
HbA1c (%)	5.8 ± 0.2	6.3 ± 0.2
epigen	274.3 ± 8.3	254.3 ± 10.3
Age (year)	48.7 ± 3.3	45.6 ± 2.4
**BMI (kg/m^2)**	22.5 ± 0.1	27 ± 0.3
SP (mmHg)	133.6 ± 2.1	134 ± 1.7
DP (mmHg)	77.9 ± 1.3	82.1 ± 1.5
Random glucose (mmol/L)	5.9 ± 0.3	7 ± 0.5
A/G	1.1 ± 0	1.2 ± 0
ALB (g/L)	52.1 ± 18.7	37.2 ± 0.9
GLB (g/L)	29.6 ± 1.4	30.7 ± 0.8
ALP (U/L)	97.6 ± 1.4	82.2 ± 5.5
ALT (U/L)	35.9 ± 5.4	41.1 ± 12.1
AST (U/L)	52.2 ± 11.1	46.5 ± 13.1
GGT (U/L)	85.8 ± 25.2	52.4 ± 9.4
DBIL (mmol/L)	20.9 ± 11.7	18.7 ± 14.2
IBIL (mmol/L)	14.5 ± 4.5	14.4 ± 4.9
TBIL (mmol/L)	33.6 ± 15	34 ± 20

Table S2 Baseline characteristics of study cohorts of normal or diabetic individuals. Data represent as mean ± SEM. Abbreviations: HbA1c, glycated haemoglobin, BMI, body mass index, SP, systolic pressures, DP, diastolic pressure, A/G, ALB/GLB ratio, ALB, albumin, GLB, globulin, ALP, alkaline phosphatase, ALT, alanine aminotransferase, AST, aspartate aminotransferase, GGT, Gamma-glutamyl transferase, DBIL, direct bilirubin, IBIL, indirect bilirubin, TBIL, total bilirubin.

	Normal	Diabetic
Gender M/F	44/24	12/8
**HbA1c (%)**	5.46 ± 0.045	8.3 ± 0.3
epigen	282.51 ± 8.59	240.1 ± 12.3
Age (year)	40.32 ± 2.29	63.1 ± 1.7
BMI (kg/m^2)	23.6 ± 0.41	25.9 ± 1
SP (mmHg)	132.31 ± 1.67	137.4 ± 3.5
DP (mmHg)	80.2 ± 1.21	81.5 ± 2
Random glucose (mmol/L)	5.46 ± 0.17	11.6 ± 1
A/G	1.04 ± 0.13	1.1 ± 0
ALB (g/L)	31.57 ± 1.74	45.3 ± 11.1
GLB (g/L)	33.4 ± 2.16	31.4 ± 1
ALP (U/L)	142.75 ± 33.47	107.9 ± 25.8
ALT (U/L)	111 ± 34.8	25.7 ± 2.6
AST (U/L)	150.58 ± 41.5	25.6 ± 3.3
GGT (U/L)	181.91 ± 54.01	99.8 ± 36.4
DBIL (mmol/L)	64.4 ± 29.04	2.8 ± 0.3
IBIL (mmol/L)	31.1 ± 9.99	7.5 ± 0.7
TBIL (mmol/L)	95.3 ± 39	10.3 ± 0.9

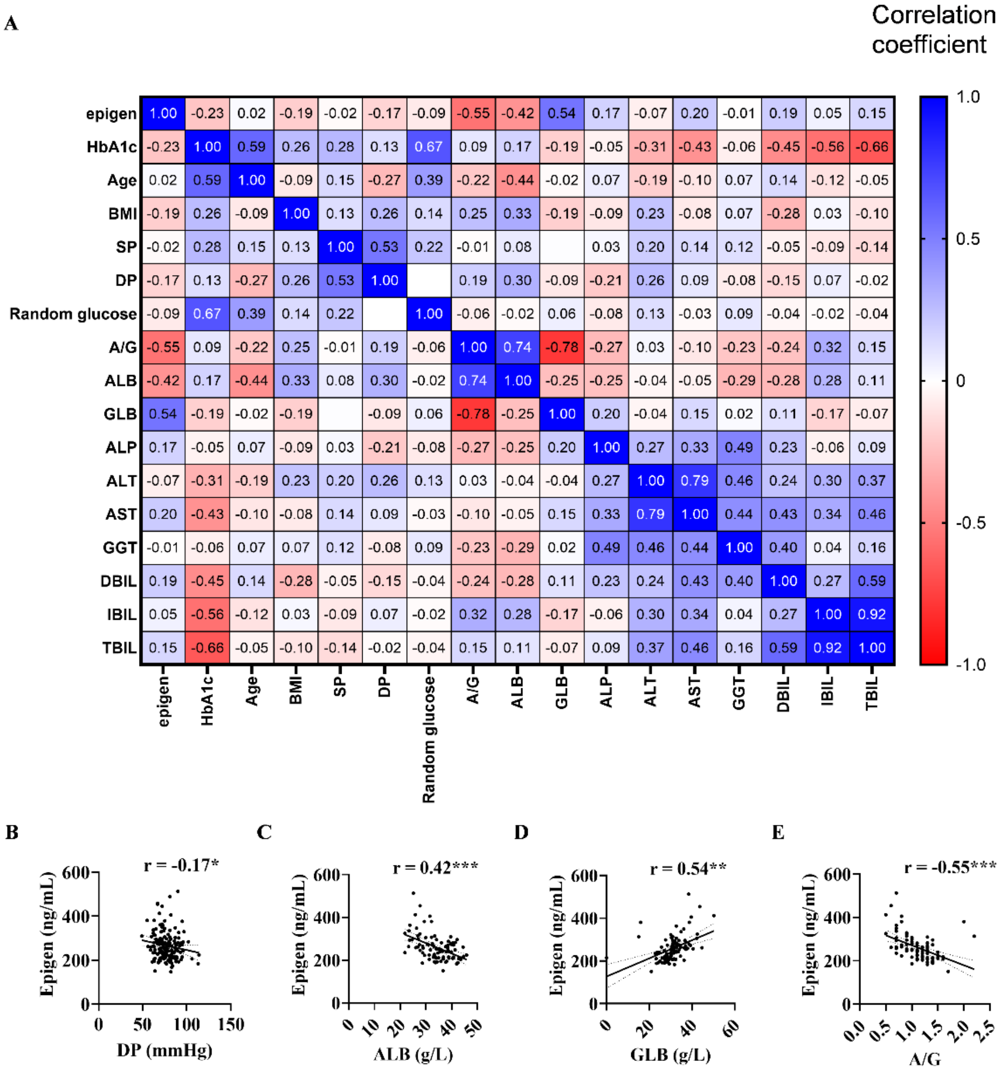

Figure S1 Correlations between circulating epigen levels and metabolic-related parameters in human serum samples. (A) Correlation matrix of epigen and metabolic-related parameters. Significant correlations between epigen and DP (B), ALB (C), GLB (D) and A/G (E). Statistical analysis was performed using Spearman's Rho Correlation. n = 159; ∗p < 0.05, ∗∗p < 0.01, ∗∗∗p < 0.001. Blank represent correlation coefficient < 0.01. Abbreviations: HbA1c, glycated haemoglobin, BMI, body mass index, SP, systolic pressures, DP, diastolic pressure, A/G, ALB/GLB ratio, ALB, albumin, GLB, globulin, ALP,

alkaline phosphatase, ALT, alanine aminotransferase, AST, aspartate aminotransferase, GGT, Gamma-glutamyl transferase, DBIL, direct bilirubin, IBIL, indirect bilirubin, TBIL, total bilirubin.

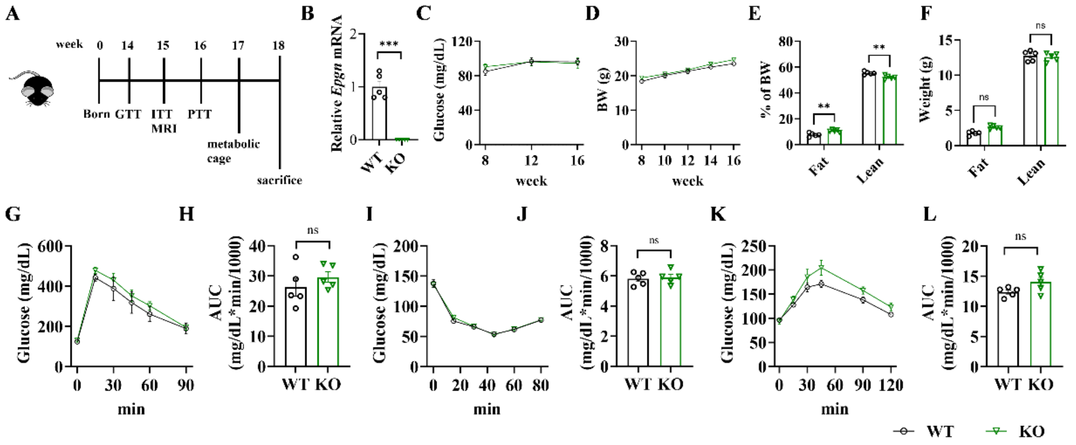

Figure S2 Glucose homeostasis in epigen knockout (KO) mice fed a standard chow diet. (A) Schematic diagram of experiment. (B) *Epgn* mRNA levels in the tongue. (C) Fasting glucose levels. Body weight measurements (D), fat and lean mass (E) and body composition (F) at 15 weeks. GTT results (G) and AUC (H) for the GTT at 14 weeks. ITT results (I) and AUC (J) for the ITT at 15 weeks. PTT results (K) and AUC (L) for the PTT at 16 weeks. n = 5 WT, 5 HFD. Data are the mean ± SEM. Statistical analysis was performed using unpaired two-tailed Student’s t-test or Welch's t-test. ∗∗∗p < 0.001.

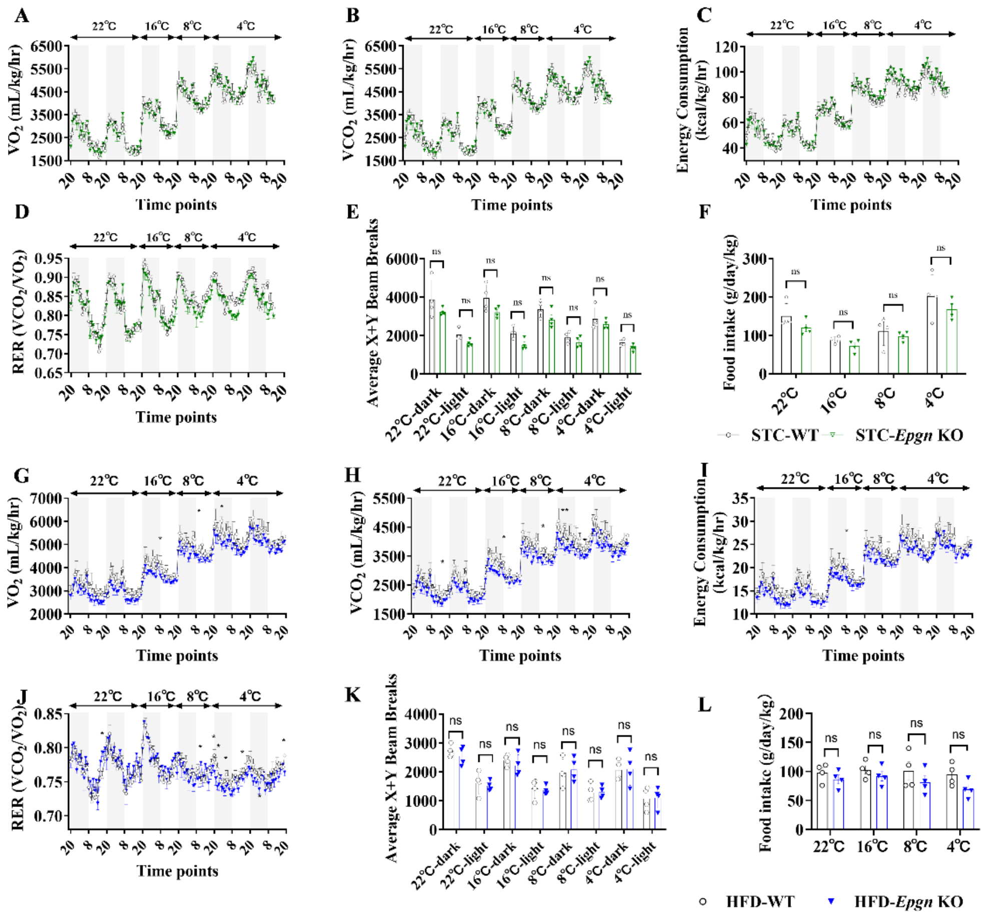

Figure S3 Energy expenditure in HFD-fed *Epgn* KO mice. Real-time monitoring curve of oxygen consumption (A), carbon dioxide release (B), energy consumption (C), respiratory exchange ratio (D) quantification of activity (E) and accumulated food intake (F) of STC-feeding 16 weeks old *Epgn* KO mice, comparing to their WT littermates. Real-time monitoring curve of oxygen consumption (G), carbon dioxide release (H), energy consumption (I), respiratory exchange ratio (J), quantification of activity (K) and accumulated food intake (L) of *Epgn* KO mice, comparing to their WT littermates after 12 weeks of HFD feeding. n = 4 WT, 4 KO. Data are the mean ± SEM. Statistical analysis was performed using unpaired two-tailed Student's t-test or Welch's t-test. $*p < 0.05$, $**p < 0.01$.

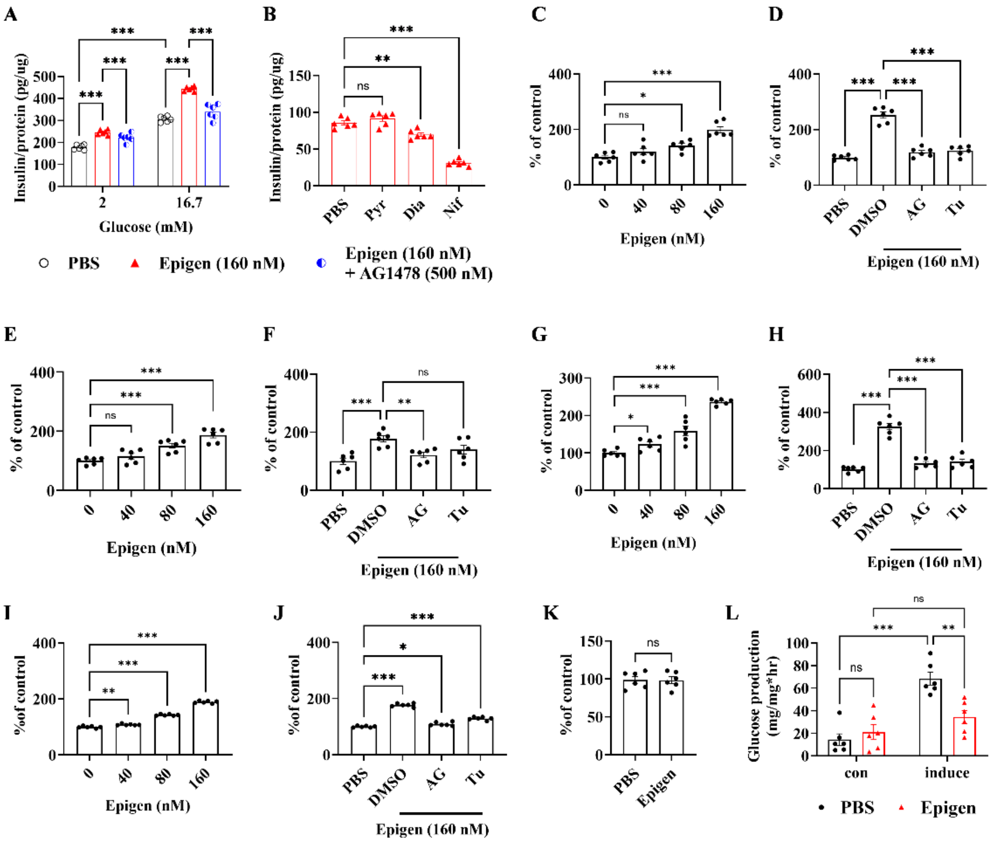

Figure S4 *In vitro* glucose uptake by cells and beta cell proliferation following acute recombinant epigen treatment. (A) Insulin secretion in SJ beta cells with epigen treatment under high glucose stimulation, with and without EGF receptor inhibitor AG1478. (B) Insulin secretion in SJ beta cells with epigen treatment, with and without calcium channel inhibitor Nifedipine (Nif). (C) Glucose uptake in 3T3-L1 differentiated adipocytes with epigen treatment in a dose-dependent manner. (D) Glucose uptake in 3T3-L1 differentiated adipocytes with and without EGF receptor inhibitors. (E) Glucose uptake in SW872 differentiated adipocytes with epigen treatment in a dose-dependent manner. (F) Glucose uptake in SW872 differentiated adipocytes with and without EGF receptor inhibitors. (G) Glucose uptake in L6 differentiated muscle cells with epigen treatment in a

dose-dependent manner. (H) Glucose uptake in L6 differentiated muscle cells with and without EGF receptor inhibitors. (I) Glucose uptake in A204 differentiated muscle cells with epigen treatment in a dose-dependent manner. (J) Glucose uptake in A204 differentiated muscle cells with and without EGFR inhibitors AG1478 and Tucatinib. (K) Glucose uptake in HepG2 cells with epigen treatment. (L) Glucose production in HepG2 cells under glucagon and pyruvate induction, with and without epigen protein treatment. Data are the mean ± SEM. Statistical analysis was performed using unpaired two-tailed Student's t-test or Welch's t-test. Group differences were determined via two-tailed analysis of variance (ANOVA) with Tukey's post hoc. $*p < 0.05$, $**p < 0.01$, $***p < 0.001$.

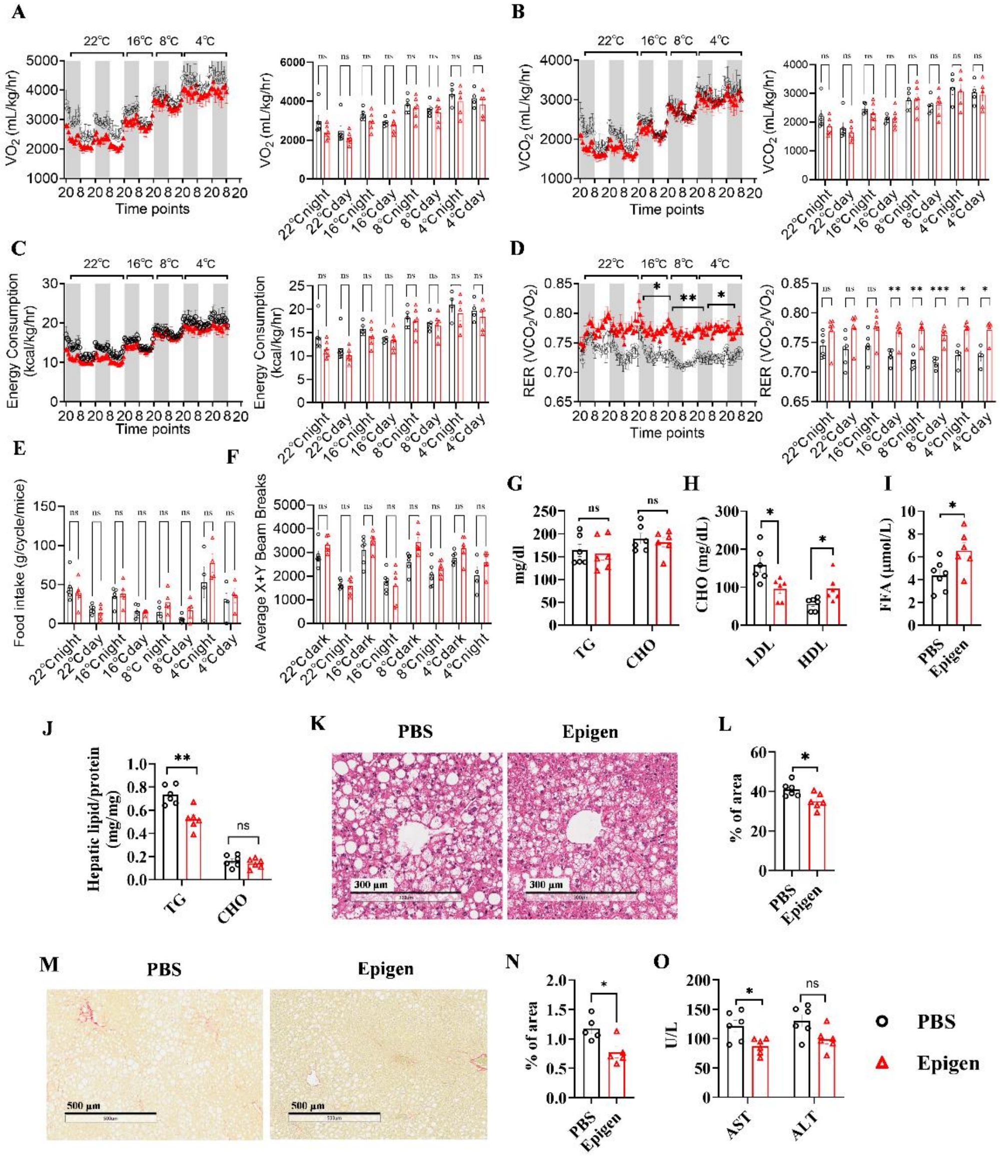

Figure S5 Energy expenditure and lipid profiles of mice after chronic epigen protein injection. (A) Real-time monitoring curve and average oxygen consumption (VO_2). (B) Real-time monitoring curve and averaged of carbon dioxide release (VCO_2). (C) Real-time monitoring curve and average energy consumption. (D) Real-time monitoring curve and average Respiratory exchange ratio ($RER = VCO_2/VO_2$). (E) Quantification of accumulated food intake. (F) Quantification of activity. (G) TG and CHO level. (H) LDL

and HDL level. (I) FFA level. (J) Hepatic lipid level. Representative images of liver H&E staining (K) and quantification of steatosis (L) Representative images of liver Sirius Red staining (M) and quantification of fibrosis (N) in liver. (O) AST and ALT levels at week 15. n = 6. Data are the mean ± SEM. Statistical analysis was performed using unpaired two-tailed Student’s t-test or Welch's t-test.

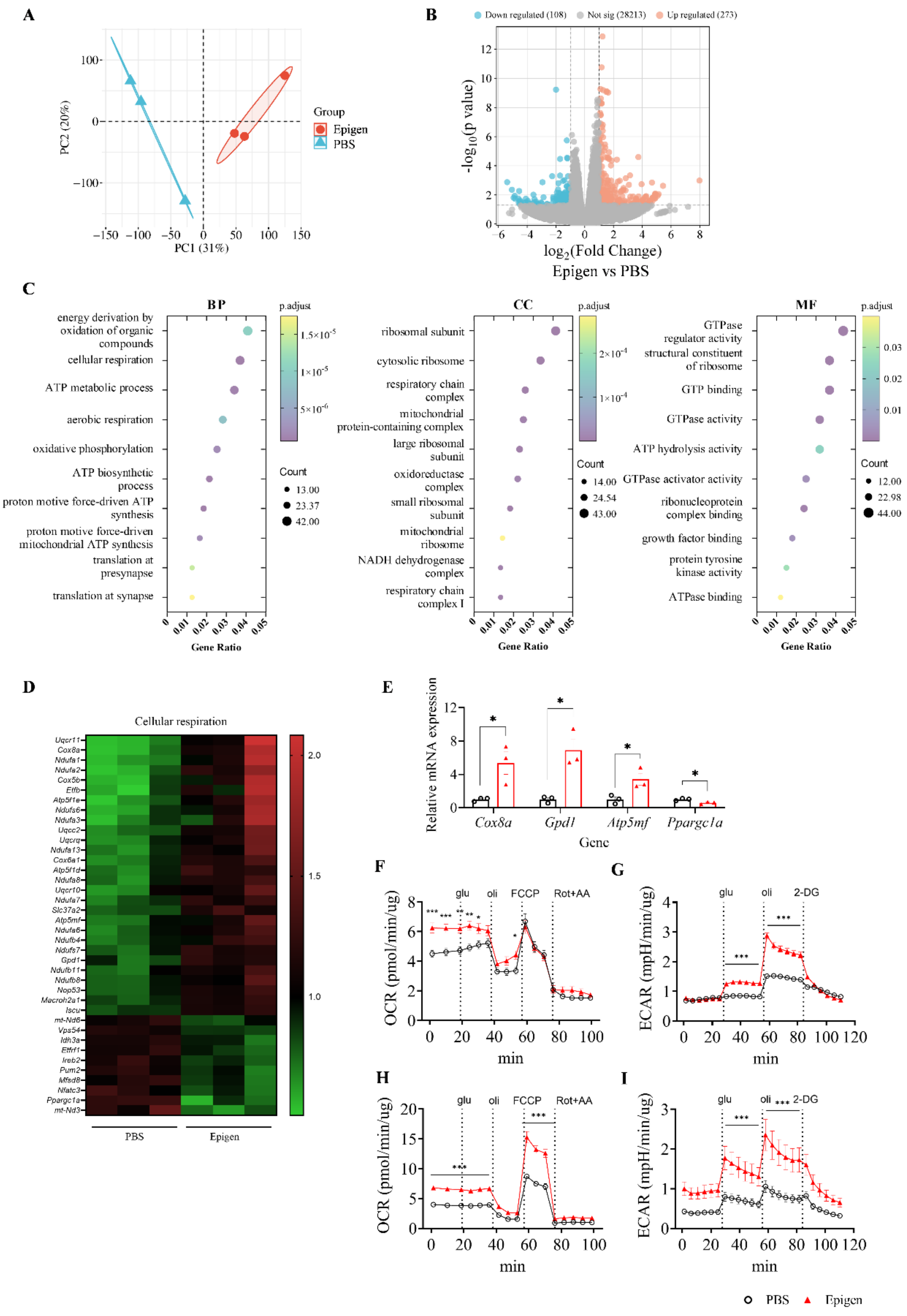

A
B
C
D
E
F
G
H
I
Group
Epigen
PBS
PC1 (31%)
PC2 (20%)
Down regulated (108)
Not sig (28213)
Up regulated (273)
Epigen vs PBS
BP
CC
MF
Gene Ratio
Count
p.adjust
Cellular respiration
Relative mRNA expression
Gene
Cox8a
Gpd1
Atp5mf
Ppargc1a
OCR (pmol/min/ug)
ECAR (mpH/min/ug)
min
glu
oli
FCCP
Rot+AA
2-DG
PBS
Epigen

Figure S6 RNA sequencing profile and functional enrichment analysis of differentially expressed genes (DEGs) in the sWAT of mice after 15 weeks of chronic epigen injection. (A) Principal component analysis (PCA) of samples. (B) Volcano plot representing sWAT DEGs. (C) Significantly enriched terms in Biological Processes (BP), Cellular Components (CC), and Molecular Functions (MF) from GO analysis. (D) Heatmap of gene expression related to the cellular respiration pathway. (E) RT-qPCR verification of the expression of select representative genes in sWAT. Measurements of oxygen consumption rate (OCR) of differentiated 3T3-L1 adipocytes (F) and differentiated SW872 adipocytes (H) with and without 160 nM epigen for 1 hour. Extracellular acidification rate (ECAR) of differentiated 3T3-L1 adipocytes (G) and differentiated SW872 adipocytes (I) with and without 160 nM epigen for 1 hour. A-E, n = 3; F, n = 8,9; G, n = 8,10; H, n = 10; I, n = 10,8. Data are the mean ± SEM. Statistical analysis was performed using unpaired two-tailed Student's t-test or Welch's t-test. $*p < 0.05$, $**p < 0.01$, $***p < 0.001$.
